# miR-99a reveals two novel oncogenic proteins E2F2 and EMR2 and represses stemness in lung cancer

**DOI:** 10.1038/cddis.2017.544

**Published:** 2017-10-26

**Authors:** Andrea Feliciano, Yoelsis Garcia-Mayea, Luz Jubierre, Cristina Mir, Manuela Hummel, Josep Castellvi, Javier Hernández-Losa, Rosanna Paciucci, Irene Sansano, Yilin Sun, Santiago Ramón y Cajal, Hiroshi Kondon, Aroa Soriano, Miguel Segura, Alex Lyakhovich, Matilde E LLeonart

**Affiliations:** 1 Biomedical Research in Cancer Stem Cells Group, Pathology Department, Institut de Recerca Hospital Vall d'Hebron (VHIR), Passeig Vall d'Hebron 119-129, 08035 Barcelona, Spain; 2 Centre for Genomic Regulation, Core Facilities - Microarray Unit, C/ Dr. Aiguader 88, 08003 Barcelona, Spain; 3 Geriatric Unit, Graduate School of Medicine, Kyoto University Hospital, Kyoto 606-8507, Japan

## Abstract

Lung cancer is one of the most aggressive tumours with very low life expectancy. Altered microRNA expression is found in human tumours because it is involved in tumour growth, progression and metastasis. In this study, we analysed microRNA expression in 47 lung cancer biopsies. Among the most downregulated microRNAs we focussed on the miR-99a characterisation. *In vitro* experiments showed that miR-99a expression decreases the proliferation of H1650, H1975 and H1299 lung cancer cells causing cell cycle arrest and apoptosis. We identified two novel proteins, E2F2 (E2F transcription factor 2) and EMR2 (EGF-like module-containing, mucin-like, hormone receptor-like 2), downregulated by miR-99a by its direct binding to their 3′-UTR. Moreover, miR-99a expression prevented cancer cell epithelial-to-mesenchymal transition (EMT) and repressed the tumourigenic potential of the cancer stem cell (CSC) population in both these cell lines and mice tumours originated from H1975 cells. The expression of E2F2 and EMR2 at protein level was studied in 119 lung cancer biopsies. E2F2 and EMR2 are preferentially expressed in adenocarcinomas subtypes *versus* other tumour types (squamous and others). Interestingly, the expression of E2F2 correlates with the presence of vimentin and both E2F2 and EMR2 correlate with the presence of *β*-catenin. Moreover, miR-99a expression correlates inversely with E2F2 and directly with *β*-catenin expression in lung cancer biopsies. In conclusion, miR-99a reveals two novel targets E2F2 and EMR2 that play a key role in lung tumourigenesis. By inhibiting E2F2 and EMR2, miR-99a represses *in vivo* the transition of epithelial cells through an EMT process concomitantly with the inhibition of stemness features and consequently decreasing the CSC population.

Lung cancer is the first leading cause of death worldwide, affecting up to 31% of men and 27% of women.^1^ Non-small-cell lung cancer (NSCLC) accounts for ∼85% of all lung cancers.^2^ Unlike other major cancers demonstrating significant improvements in survivability, the 5-year survival rate for lung cancer has remained constant at ~15%. This lack of improvement could be because of the high degree of histological heterogeneity of lung tumours, the difficulties in early diagnosis and the inability to rapidly assess therapeutic effects.^3^

The microRNAs have been shown to play an important role in many biological processes, including cellular proliferation.^4, 5, 6^ Several microRNAs deregulated in cancers have been found to target tumour-suppressor genes/oncogenes that play a role in cellular transformation.^7, 8^

In this study, we screened microRNA expression levels in patients with NSCLC using microarrays. We shortlisted microRNAs whose expression patterns were significantly different between normal and cancer tissues. Among the most downregulated microRNAs, we focussed on miR-99a that has been reported to be deregulated in NSCLC and renal cell carcinoma.^9, 10^ miR-99a has been associated with the cancer stem cell (CSC) population in a model of breast cancer but its role in lung CSCs remained unknown.^11^ Here, we describe two novel targets of miR-99a, E2F2 (E2F transcription factor 2) and EMR2 (EGF-like module-containing, mucin-like, hormone receptor-like 2), and their association with epithelial-to-mesenchymal transition (EMT) repression and expression of stem cell genes.

## Results

### A microRNA signature distinguishes normal from tumour tissue in NSCLC patients

Results of the analysis from the microRNA array containing the initial series of 24 patients are shown in Supplementary Table 1. We observed significant differences in 97 out of 799 microRNAs when comparing normal *versus* tumour tissues (Supplementary Table 2). Based upon the differential expression patterns of the 97 microRNAs, all 48 samples (24 normal and 24 tumour) were clustered by similarity into subgroups without using any information regarding the identity of the samples. Samples were divided into normal and cancer groups based on the whole list of microRNAs contained in platform 1 (Supplementary Figure 1a). In a few cases some tumours were clustered in the healthy group, and in one case healthy tissue was clustered in the tumour group. By microRNA signature, we define the list of microRNAs that are differentially expressed in tumours *versus* normal tissues. In order to find a microRNA signature enabling patient subgrouping, patients were clustered based on the tumour/normal expression ratios of the 97 selected microRNAs (Supplementary Table 2). Significant association between the resulting clusters with tumour type and the degree of tumour differentiation was found (Supplementary Figures 1b and c).

No other associations were found between the clusters and various clinicopathological features, including age, sex, patient status or disease-free survival, according to the microRNA expression pattern analysis.

**Figure 1:**
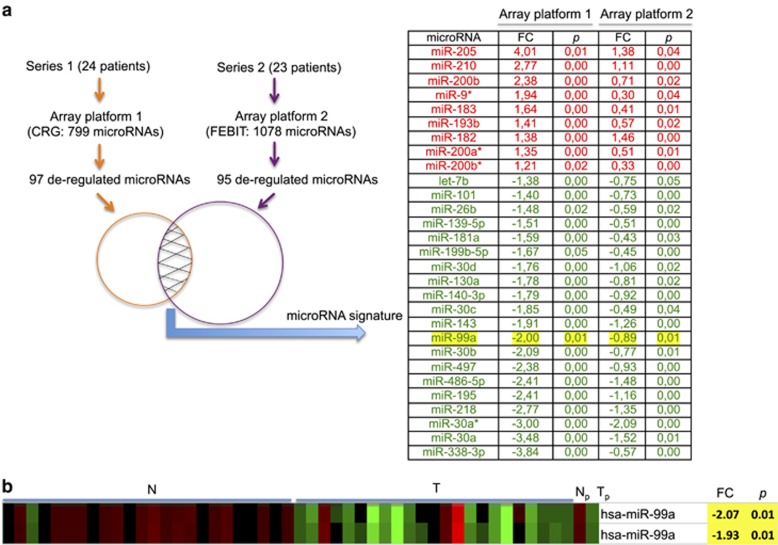
MicroRNA array study. (**a**) Schematic representation of the methodology to identify a microRNA signature able to distinguish healthy from tumour tissue in NSCLC patients is shown. The signature comprises 29 microRNAs: 9 upregulated (red) and 20 downregulated (green) in comparison with normal tissue. (**b**) Array results for the miR-99a that was chosen out of the whole deregulated microRNAs for future experiments. N, normal tissue; T, tumour tissue; N_p_, pool of normal tissues; T_p_, pool of tumour tissues; FC, fold change, *P* (*T*-test value)

In order to identify microRNAs useful as biomarkers to differentiate subtypes of NSCLC, we studied the correlation of each differentially expressed microRNA (Supplementary Table 2) with the histological type. The only microRNA able to distinguish cancer subtypes was miR-205. Other microRNAs, including miR-101, miR-101*, miR-181a, miR-30b and miR-338-3p, demonstrated correlation with the differentiation status of the tumours (Supplementary Figures 2a and b). miR-99a was among of the most downregulated microRNAs (Supplementary Table 2). In order to verify the results from the array, a total of 10 patients from series 1 were studied for the expression of miR-99a by qRT-PCR (Supplementary Figures 3a and b). Results from the qRT-PCR corroborate well the data from the microRNA array for assessing up- or down-regulated miR-99a. Moreover, an independent series of patients (series 2) was studied for the whole microRNA profile (Supplementary Table 3). A total of 95 deregulated microRNAs were identified in this second series of 23 patients (48 upregulated and 47 downregulated), of which 29 microRNAs were common with the first series and miR-99a was also confirmed as one of the most downregulated microRNAs (Figure 1a and Supplementary Table 4). Such 29 deregulated microRNAs have the power to distinguish tumour *versus* normal tissue. A common signature was established for lung cancer tumours (Figure 1a). For further experiments, we focussed on the characterisation of miR-99a (Figure 1b).

### miR-99a reduces the proliferative capacity of NSCLC cells

**Figure 2:**
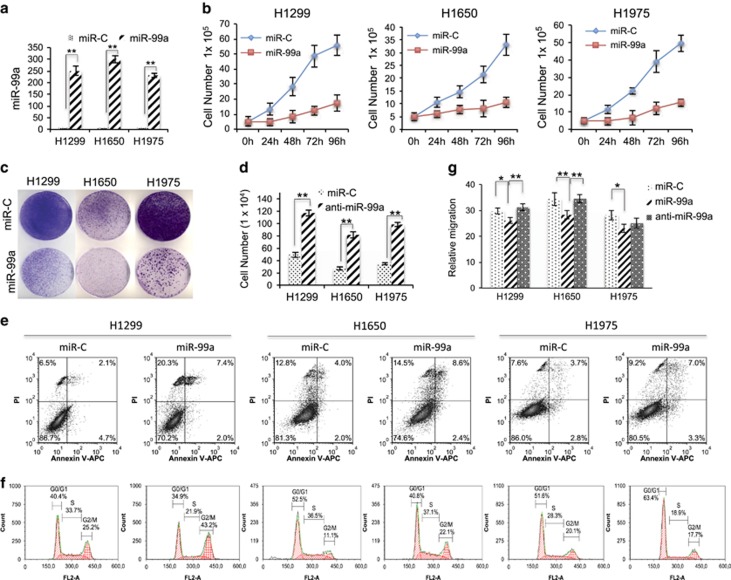
miR-99a exerts a tumour-suppressor function in lung cancer cells. (**a**) Relative qRT-PCR shows the expression levels of NSCLC cell lines upon miR-99a transient expression. (**b**) Proliferation curves of miR-99a-expressing cells *versus* control cells (transfection time was considered 0 h). (**c**) Cristal violet-stained colonies indicative of cell proliferation of miR-99a-expressing cells and controls after 48 h upon transfection. (**d**) Cell number of the indicated cells transfected with an anti-miR-99a or control (miR-C) microRNA. (**e**) Plots of miR-99a-expressing cells and controls stained with Annexin-V indicative of apoptotic cells and analysed by FACS. Note that miR-99a increases late apoptosis (right upper panel) in all cases. (**f**) Cell cycle profile of miR-99a cells *versus* controls in the indicated cell lines. Note that miR-99a arrests cells in G2 phase of the cell cycle in H1299 and H1650 cell lines and arrests cells in G1 in H1975 cells. PI, propidium iodide. Data are representative of at least three independent experiments. (**g**) Relative migration of H1299, H1650 and H1975 cells transduced with miR-99a and anti-miR-99a. (*P<0.05; **P<0.01)

To assess the properties of miR-99a, the H1299, H1650 and H1975 cells were transduced with miR-99a mimic (miR-99a) or miR-C (non-target control). miR-99a expression was verified by qRT-PCR (Figure 2a). Transduction with miR-99a decreased proliferation of NSCLC cells (Figures 2b and c and Supplementary Figure 4a). Proliferation profiles of either stably or transiently miR-99a-expressing cells were similar (Supplementary Figures 4b–d, data not shown). The suppressive function of miR-99a was maintained at physiological levels, as shown by treatment of the above cells with 1:10 and 1:3 diluted miR-99a-viral supernatants (Figure 2 and Supplementary Figures 5 and 6a). Conversely, anti-miR-99a was able to reverse proliferative effect of miR-99a (Figure 2d, data not shown).

### miR-99a suppresses tumourigenicity by inducing apoptosis and cell cycle arrest

To uncover possible mechanisms of the miR-99a-mediated suppression of cell proliferation, cell cycle arrest, apoptosis and senescence were studied. An increase in apoptosis was detected after miR-99a expression in the three cell lines (Figure 2e). This was accompanied by cell cycle arrest in miR-99a-expressing cells as compared with control cells (Figure 2f). H1299 and H1650 accumulated in the G2 phase and H1975 in the G1 phase of the cell cycle. Accordingly, a decrease in the number of miR-99a-expressing cells was confirmed by Trypan-blue staining (Supplementary Figure 4e). miR-99a-expressing cells were negative for *β*-galactosidase staining, discarding the possibility of cell senescence (data not shown). Moreover, cells infected with miR-99a viral construct in a 1 : 10 dilution were also able to increase apoptosis (Supplementary Figure 6b, data not shown). Overall, a tumour-suppressor function of miR-99a was observed upon expression of miR-99a in all lung cancer cell lines.

### E2F2 and EMR2 are revealed as two novel miR-99a targets

**Figure 3:**
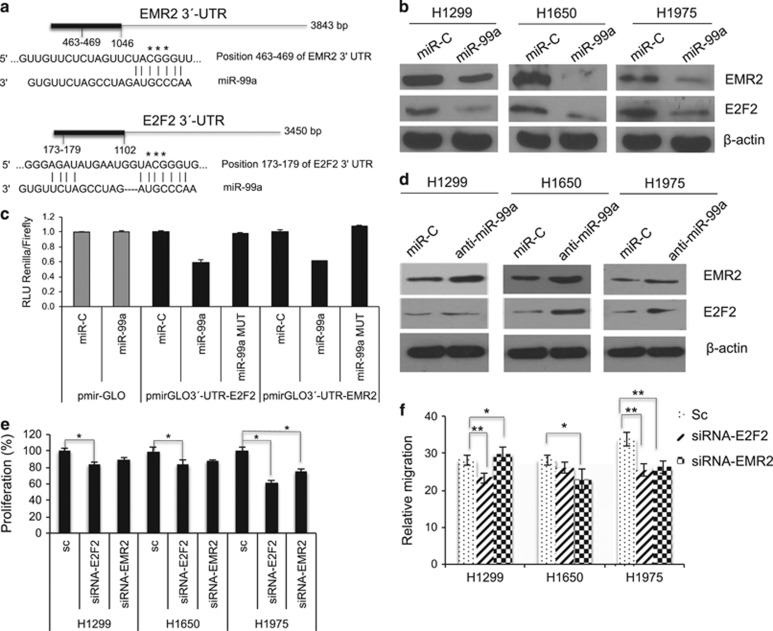
E2F2 and EMR2 are downregulated upon miR-99a expression. (**a**) Schematic representation indicating the cloned region of the 3′-UTR of EMR2 (1046 bp) and E2F2 (1102) mRNA. Positions of the binding regions of miR-99a (corresponding to the seed sequence) to each 3′-UTR are indicated (463–469 and 173–179, respectively). The asterisks in the 3′-UTR region indicate the changes introduced in the DNA sequence for the design of each respective mutant. (**b**) Western blots showing the protein levels of E2F2 and EMR2 in miR-99a-expressing cells in the indicated cell lines. *β*-Actin is shown for protein content. (**c**) Luciferase reporter experiment for the miR-99a-expressing cells concomitantly with the pmirGLO plasmid (control), the pmirGLO3′-UTR-E2F2 or the pmirGLO3′-UTR-EMR2. Relative expression of *Renilla versus* Firefly (luciferase) expression is shown. Note that miR-99a is able to decrease the expression of both 3′-UTRs. (**d**) Western blots of E2F2 and EMR2 in anti-miR-99a-expressing cells in the indicated cell lines. Data are representative of at least three independent experiments. (**e**) Cell number of H1299, H1650 and H1975 cells transduced with the indicated siRNAs and counted upon 48 h from transfection. (**f**) Relative migration of H1299, H1650 and H1975 cells transduced with the indicated siRNAs. (*P<0.05; **P<0.01)

Bioinformatic search revealed two potential novel targets of miR-99a, EMR2 and E2F2. A schematic representation of the binding sites of miR-99a with EMR2 and E2F2 is shown (Figure 3a). Downregulation of both EMR2 and E2F2 proteins occurred upon expression of miR-99a (Figure 3b and Supplementary Figure 7a). To validate these proteins as miR-99a targets, the 3′-UTR of each gene was cloned in the pmirGLO vector (Figure 3a). Both the pmirGLO 3′-UTR-E2F2 and pmirGLO 3′-UTR-EMR2 plasmids were transfected in HEK293T cells concomitantly with miR-99a. The presence of miR-99a was able to inhibit the expression of each wild-type 3′-UTR and the 3′-UTR mutants revealed no effects, indicating that these two proteins are targets of miR-99a (Figure 3c). Moreover, the effect of an anti-miR-99a was accompanied by an increase of EMR2 and E2F2 protein expressions (Figure 3d and Supplementary Figure 7b). The contribution of E2F2 and EMR2 to cell proliferation was tested in siRNA depletion experiments (Supplementary Figure 7d). The depletion of E2F2 and/or EMR2 in H1299, H1650 and H1975 cells reduced cell proliferation (Figure 3e). Overall, these data provide compelling evidence of two novel targets for miR-99a.

### miR-99a suppresses invasion and migration whereas favours adhesion of NSCLC cells

Two previous publications described the ability of miR-99a to reduce migration and invasion in bladder and lung cancer cells.^12, 13^ In order to address whether miR-99a reduces migration and invasion of H1299, H1975 and H1650 cells upon transduction with miR-99a, both transwell mobility and wound healing assays were performed. miR-99a reduced the migration and invasion in all three lines (Figure 2g, Supplementary Figures 8a and 9a–b, data not shown). Moreover, miR-99a overexpression stimulated cell adhesion (Supplementary Figure 8b, data not shown). In order to validate the miR-99a biological effect, the anti-miR-99a was included for comparison (Figures 2g and 8a and Supplementary Figure 9a). In addition, for the above three cell lines a partial contribution of E2F2 and EMR2 to migration, invasion and adhesion was confirmed by siRNA experiments (Figure 3f and Supplementary Figures 8c–d and 9c). These results support the tumour-suppressor function of miR-99a and its link with the identified targets.

### E2F2 and EMR2 overexpression is concomitant to miR-99a

**Figure 4:**
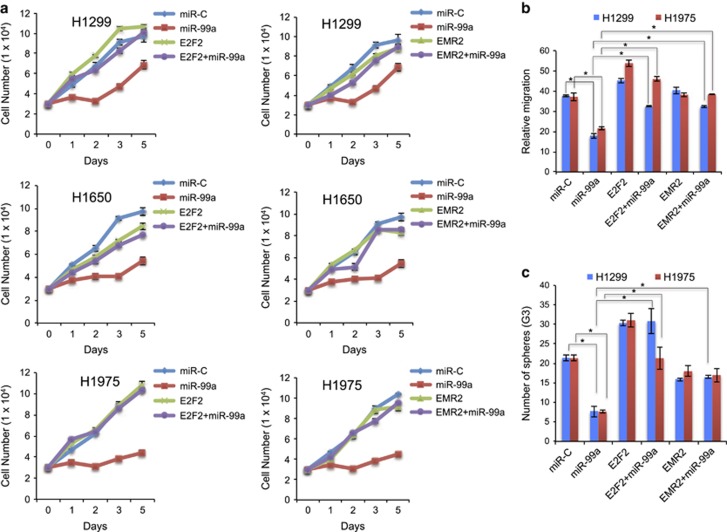
E2F2 and EMR2 gene overexpression concomitantly to miR-99a. (**a**) Proliferation curves of the indicated cancer cell lines upon 48 h from transfection with the indicated genes and/or miR-99a. Right panel corresponds to EMR2 and left panel corresponds to E2F2-expressing cells (with and without miR-99a). Note the total proliferative rescue upon E2F2 expression and lack of rescue of EMR2 under the same conditions. (**b**) Relative migration of H1299 and H1975 cells transduced with miR-99a and E2F2 or EMR2. Significant increase in migration occurs upon overexpression of E2F2 and EMR2 concomitantly to miR-99a when compared with miR-99a alone (plus empty vector). (**c**) Number of spheroids from third-generation (G3) cells growing in stem cell media and nonadherent dishes upon overexpression of E2F2 and EMR2 concomitantly to miR-99a *versus* controls. (*P<0.05)

In order to analyse whether E2F2 or EMR2 expression was able to rescue the suppressive function of miR-99a, coexpression of E2F2 or EMR2 genes was performed concomitantly with miR-99a (Figure 4a). We demonstrate that E2F2 but not EMR2 was able to rescue the suppressive function of miR-99a in H1299, H1650 and H1975 cells (Figure 4a). We also found that concomitant expression of E2F2 and EMR2 with miR-99a was able to rescue the inhibitory role of miR-99a on cell migration (Figure 4b). Importantly, E2F2 showed a potent effect on cell migration as the sole expression of E2F2 increased migration in comparison with controls (miR-C).

### miR-99a suppresses tumourigenicity *in vivo*

**Figure 5:**
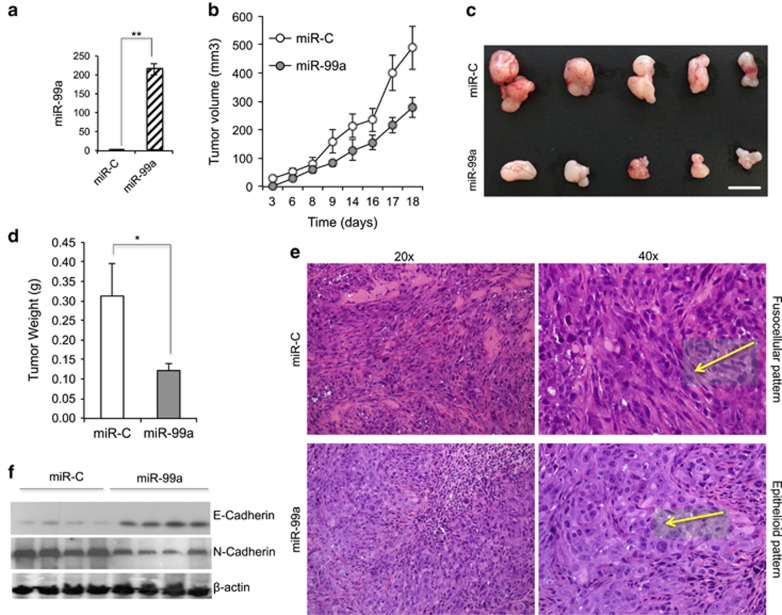
miR-99a expression delayed tumour formation *in vivo*. (**a**) H1975 cells were transiently transduced with miR-99a and measured for miR-99a RNA levels by qRT-PCR before injecting in mice. (**b**) Graph showing tumour volume in miR-99a-expressing tumours *versus* controls. (**c**) Representative pictures of tumours from mice transduced with miR-99a *versus* controls. (**d**) Graph showing tumour weight of miR-99a-expressing tumours *versus* controls. (**e**) Example of phenotypic morphology of mice tumours (H&E staining) expressing miR-99a or control microRNA. Arrows indicate the fusocellular pattern (control group) or epithelial pattern (miR-99a group) in each case. (**f**) Western blots showing expression of N-cadherin and E-cadherin for the indicated group of mice tumours. (*P<0.05; **P<0.01)

To confirm the tumour-suppressive function of miR-99a *in vivo*, 1 × 10^6^ miR-99a-expressing H1975 cells and controls were xeno-injected subcutaneously to immunocompromised mice. The levels of miR-99a and target proteins were verified after transfection and just before the injection (Figure 5a, data not shown). Tumours formed by miR-99a were smaller than those formed in the control group (Figures 5b–d). The microscopic examination of tumours revealed a consistent pattern of heterogeneous tumours with fusocellular morphology in the control group in contrast to a more homogeneous epithelial pattern observed in tumours overexpressing miR-99a (Figure 5e). The morphology of the control tumours resembled the EMT transition associated with more aggressive tumour phenotypes.^14^ Increased protein expression levels of N-cadherin and decreased E-cadherin expression, as indicators of the EMT, were observed in the mice tumours derived from the control group but not in those originated from miR-99a expression (Figure 5f). Moreover, changes in protein expression of N-cadherin and E-cadherin were also observed in H1975 cells expressing miR-99a *versus* control cells (Supplementary Figure 10a).

**Figure 6:**
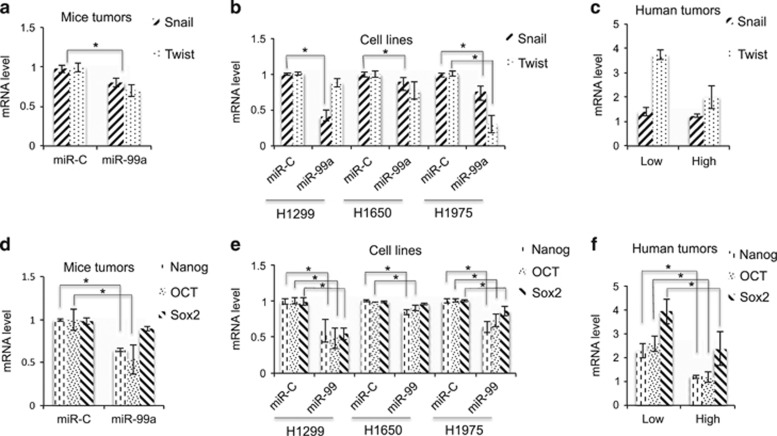
Relative mRNA levels of genes related to stemness. Snail and Twist genes were analysed by qRT-PCR in the mice tumours (**a**), NSCLC cell lines (**b**) and human tumours (**c**). Nanog, Oct3/4 and Sox2 genes were analysed by qRT-PCR in the mice tumours (**d**), NSCLC cell lines (**e**) and human tumours (**f**). (**c** and **f**) From the array results (Supplementary Table 1), top 4 cancers with higher expression of miR-99a were selected (designated as ‘High’), and the 4 cancers with the lowest expression of miR-99a (designated as ’Low’) are depicted in the graph (*P<0.05)

Other EMT-related genes, such as Snail and Twist, were downregulated in the miR-99a-expressing mice tumours and NSCLC cells but not in biopsies (Figures 6a–c). In addition, downregulation of the stem cell genes Nanog, Oct3/4 and Sox2 were consistently observed in the mice tumours, NSCLC cells and biopsies (Figures 6d–f).

### miR-99a expression targets CSCs

**Figure 7:**
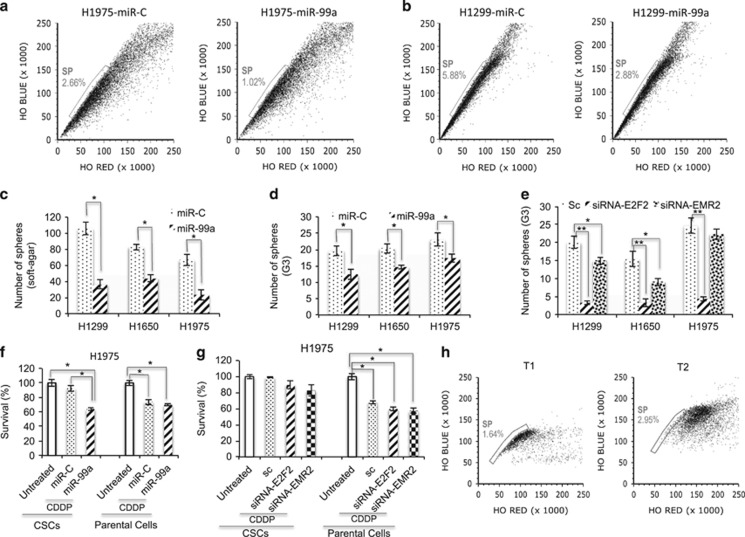
miR-99a modulates stemness. (**a** and **b**) Plots from the FACS analysis of the SP cells representative of CSCs in miR-99a-expressing cells *versus* controls in the indicated cell lines (H1975 and H1299, respectively). Note the decrease in SP in miR-99a-expressing cells after 48 h upon transfection. (**c**) Number of spheroids from the indicated cells expressing miR-99a or control microRNA growing in soft agar (as representative of three-dimensional culture). (**d**) Number of spheroids from third-generation (G3) CSCs growing in stem cell media and nonadherent dishes in miR-99a-expressing cells *versus* controls. (**e**) Number of spheroids from third-generation (G3) CSCs (growing as in Figure 6d) in the indicated cells depleted for E2F2 and EMR2 *versus* controls. (**f**) Survival of CSCs (growing as in Figure 6d) and parental cells to CDDP treatment in miR-99a-expressing cells and controls. (**g**) Survival of CSCs (growing as in Figure 6d) and parental cells to CDDP treatment in cells depleted for E2F2 and EMR2. Data are representative of at least three independent experiments. (**h**) Example of two lung cancer biopsies for the analysis of the percentage of SP cells. See RNA levels of miR-99a for comparison (Supplementary Figure 8c). T_1_, tumour 1; T_2_, tumour 2 (*P<0.05; **P<0.01)

As the CSC-related genes were downregulated in the mice tumours, NSCLC cells and biopsies, we hypothesised that miR-99a might play an important role in acquisition of CSC features in lung tumourigenesis. In order to detect cells with a stem cell-like properties, the ‘Side Population’ (SP) discrimination assay has been performed for H1975 cells expressing miR-99a or miR-C. The percentage of the SP detected in control or miR-99a expressing H1975 cells was 2.66% and 1.02%, respectively, suggesting increased stem-like cancer cells (Figure 7a). Expression of miR-99a also decreased two times the SP number in H1299 cells (Figure 7b). We then studied contribution of E2F2 and EMR2 proteins to the formation of SP. Inhibition of E2F2 and EMR2 resulted in the decrease of the SP in H1975 and H1299 cells (Supplementary Figures 11a and b). In order to validate the biological activity of the remaining SP, three different functional assays were performed. First, NSCLC cells that normally grow in standard adherent conditions were transfected with miR-99a and forced to grow in tridimensional cultures (soft agar), allowing to form colonies during 10–15 days. The miR-99a-expressing cells formed fewer colonies than control cells (Figure 7c). Second, NSCLC cells were transfected with miR-99a in nonadherent conditions with a stem cell media. NSCLC spheres were dissociated and reseeded again up to three times (G3). Evidence that the spheroid cells hold CSC properties was demonstrated by increase expression of stem cell genes ALDH1, Nestin, Sox4 and Oct4 in comparison with the same cells growing in adherent conditions (Supplementary Figure 10b). Under these conditions, miR-99a-expressing CSCs formed fewer colonies than those CSCs derived from control cells (Figure 7d). In order to observe whether E2F2 and EMR2 were involved in the ability of CSCs to form spheres, we performed siRNA experiments. The depletion of either protein decreased the ability of corresponding NSCLC cells to form spheres with siRNA-E2F2 showing a major effect (Figure 7e). Concomitant expression of E2F2 or EMR2 with miR-99a restored the effect of sphere formation (Figure 4c). At third, miR-99a-expressing spheroid CSCs, adherent (parental) or control cells were grown in the presence of cisplatin (CDDP) and cell viability was measured at 48 h post treatment. CSCs revealed higher resistance to CDDP than their corresponding parental counterparts (Figure 7f). Moreover, miR-99a expression sensitised CSCs to the exposure of CDDP but had no effect on the parental H1975 cells (Figure 7f). Corresponding treatment with CDDP of the cells depleted for either EMR2 or E2F2 led to sensitisation of the parental cells but not CSCs (Figure 7g). Finally, representative lung cancer biopsies were assessed for the SP and miR-99a expression. An inverse correlation with miR-99a level was found (Figure 7h and Supplementary Figure 11c). Overall, the above results suggest that miR-99a reverses the phenotype of CSCs by decreasing their tumourigenic potential.

### E2F2 and EMR2 proteins are expressed in a subset of lung cancer patients

**Figure 8:**
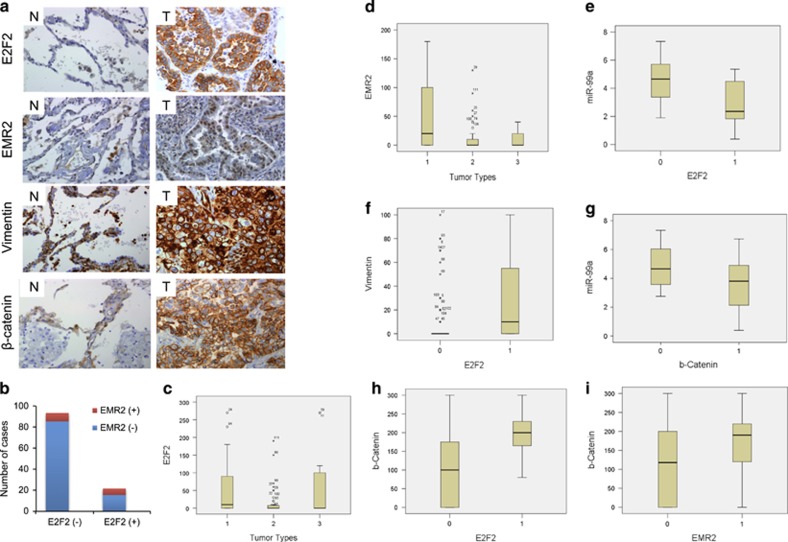
E2F2, EMR2, vimentin and *β*-catenin expression as revealed by IHC. (**a**) Representative pictures of the staining of the indicated proteins in lung cancer patients. (**b**) Significant correlation between E2F2 and EMR2 (*P*=0.026). (−) Negative expression for the indicated proteins; (+) positive expression for the indicated proteins. (**c** and **d**) Correlation of E2F2 and EMR2 with adenocarcinoma tumours (1, adenocarcinoma; 2, squamous cell carcinoma; 3, others uncommon tumour types from the lung such as neuroendocrine, and so on) (*P*=0.003 and *P*=0.01, respectively). (**e**) Significant correlation of miR-99a and E2F2 protein expression (*P*=0.025). (**f**) Significant correlation of E2F2 and vimentin protein expression (*P*=0.003). (**g**) Significant correlation of miR-99a and *β*-catenin protein expression (*P*=0.035). (**h** and **i**) Significant correlation of E2F2 and EMR2 with *β*-catenin protein expression in the 119 patients analysed (*P*=0.001 and *P*=0.032, respectively)

In order to test whether E2F2 and EMR2 are involved in lung cancer progression, immunohistochemistry (IHC) analysis from 119 patients was performed (Figure 8a and Supplementary Table 5). Expression of E2F2 and EMR2 was detected in 18% and 11.7% of patients respectively. Concomitant expression of E2F2 and EMR2 occurred in a subset of lung cancer samples (Figure 8b). E2F2 and EMR2 expression was observed predominantly in the patients with adenocarcinoma rather than with squamous cell carcinoma (Figures 8c–d). To determine whether E2F2 and/or EMR2 expression was able to inversely correlate with miR-99a expression in patients, RNA was extracted from a group of 30 randomly taken patients out of 119. The expression level of E2F2 (but not EMR2) inversely correlated with miR-99a expression (Figure 8). To determine whether the expression of E2F2 and EMR2 proteins correlated with the presence of the EMT, vimentin expression was analysed (Figure 8a and Supplementary Table 5). E2F2 expression correlated with vimentin expression (Figure 8f). Moreover, the expression levels of *β*-catenin were studied as its potential link with Wnt pathway activation,^15, 16^ known to be active in CSCs (Figure 8a and Supplementary Table 5). Expression of *β*-catenin inversely correlated with miR-99a expression (Figure 8g). In addition, *β-*catenin expression correlated with the expression of E2F2 and EMR2 (Figures 8h and i). These results suggest that overexpression of E2F2 and potentially EMR2 can be associated with lung cancers that would pursue through an EMT with potential activation of stemness.

## Discussion

In this study, a microRNA signature that revealed microRNA candidates of oncogenic and tumour suppressor functions in lung cancer is proposed. The results from the array indicate that cancer can be distinguished from healthy tissue based on the microRNA expression profile. Particularly, miR-99a could be a potential therapeutic marker in lung cancer.^17^ We described two novel miR-99a targets, E2F2 and EMR2, representing two oncogenic proteins that could modulate tumour suppression in NSCLCs. Downregulation of these proteins by miR-99a provokes apoptosis and cell cycle arrest with a consequent decrease of proliferative capacity. We found that miR-99a-mediated decrease of cell proliferation elicits a different response depending on the cell line. Thus, G2/M cell cycle arrest is induced in H1299 and H1650 cells, and G0/G1 cell cycle arrest is induced in H1975 cells. This finding highlights the functional plasticity of miR-99a according to the cellular context. Our results support previous studies reporting a tumour-suppressive function for miR-99a as a general mechanism for other cancer models.^18^

E2Fs members represent a family of transcription factors involved in a myriad of functions, including the control of cell cycle. However, the specific function of E2F2 is contradictory.^19, 20, 21, 22, 23, 24^ EMR2, a member of the EGF-TM7 receptor family, is a cell surface receptor involved in cell migration and trafficking.^25, 26^ Interestingly, a significant but low number of colorectal carcinomas are positive for EMR2 and EMR2 is relevant in breast cancer progression.^27, 28^ In NSCLC cells, we found that the action of both E2F2 and EMR2 concurred to the suppressor function of miR-99a, thereby supporting a proliferative and pro-oncogenic role for these proteins. Suppression of E2F2 and EMR2 reduced proliferation, migration and invasion and increased adhesion of NSCLC cells. Moreover, depletion of E2F2 and EMR2 reduced the number of colonies in three-dimensional cultures. Accordingly, E2F2 but not EMR2 overexpression concomitantly to miR-99a was able to rescue the suppressive function of miR-99a in proliferation of NSCLC cells. Instead, both E2F2 and EMR2 were able to partially rescue cell migration and the ability to form spheroids. We conclude that the major contributor of the suppressive function of miR-99a in NSCLC cells is E2F2 and the contribution of EMR2 is only partial. This is in agreement with the described role of EMR2.^27, 29^ The minor effect of EMR2 could be related to its multiple isoforms with unknown contribution of each one to the final function of the protein (we used the isoform 1).

The studies of E2F2 with EMT, previously associated with metastases and chemoresistance,^30, 31^ are contradictory.^20, 32, 33^ In the current study, we found that all mice tumours formed by miR-99a overexpression showed a fusocellular pattern different from the tumours formed in the control group, supporting a role of miR-99a in EMT inhibition concomitant to a downregulation of stem cell genes. miR-99a levels in the mice tumours were able to persist upon few weeks from the initial transient transfection in H1975 cells (Supplementary Figure 11d). Of note, lung cancer biopsies with high expression levels of miR-99a showed downregulation of the stem cell genes when compared with biopsies with very low expression. The consistency of the downregulation of the stem cell genes (cells, mice tumours and human biopsies) led us to hypothesise that miR-99a could modulate the CSC population. Indeed, cells overexpressing miR-99a have less number of CSCs and less self-renewal ability that are known characteristics of CSCs. Our results support a recent study showing that E-cadherin repression increases the amount of CSCs in A549 cells.^34^ Therefore, activation of the EMT can increase CSC number and that miR-99a can inhibit the viability of the CSC population. Moreover, miR-99a exerts a tumour-suppressor function not only in CSCs (decreasing their percentage and functionality) but also in parental cells from the lung (decreasing their proliferation capacity). In particular, CSC sensitisation to CDDP by miR-99a is not only due to the inhibition of E2F2 and/or EMR2 but also due to participation of other proteins.

We also found a positive correlation between the expression of E2F2 and EMR2 in human tumours. In such tumours, miR-99a expression inversely correlated with E2F2 expression. Particularly, in adenocarcinomas E2F2 expression correlated with EMT marker vimentin.

Activation of Wnt/*β*-catenin pathway has been associated with metastasis and proliferation.^35, 36^ Differential *β*-catenin expression levels and localisation are associated with cancer prognosis.^37^ We observed that: (1) miR-99a expression inversely correlated with *β*-catenin expression and (2) E2F2 and EMR2 expression correlated with *β*-catenin expression in the lung cancer biopsies. This association supports the results obtained from the mice tumours that pointed out that lower expression of E2F2 and EMR2 proteins (due to miR-99a upregulation) favours an epithelial phenotype and downregulation of stemness-associated genes.

To our knowledge, this is the first study that associates the tumour-suppressor function of miR-99a with E2F2 and EMR2 repression. Its action is accompanied by a decrease of EMT and downregulation of stem cell genes *in vivo*. In view of our data, we propose that those lung cancer tumours with high miR-99a levels and corresponding repression of E2F2 and EMR2 would evolve more favourably because of an inhibition of cell proliferation. This process is accompanied by avoiding epithelial cancer cells through an EMT process that would render higher number and functionality of CSCs.

## Materials and methods

### Patients

Forty-seven paired samples of human NSCLC and their matched adjacent noncancerous tissues were collected at the time of surgery between 2008 and 2010 from the Tumour Bank of the Pathology Department of the Hospital Vall d'Hebron. The matched normal tissues were obtained 5 cm from the tumour margin, which were further confirmed by pathologists. Upon resection, human surgical specimens were immediately frozen in liquid nitrogen and stored at −80 °C in the refrigerator. Patients recruited for this study did not undergo any therapy before resection. Series 1 contained 24 patients and series 2 contained 23 patients. Series 1 includes: 11 adenocarcinomas, 7 large cell and 6 squamous cell lung carcinomas. The characteristics of the patients from series 1 are shown (Supplementary Table 6). Series 1 was used for screening of the microRNA expression profiling using the facility from the Centre for Genomic Regulation (CRG platform, version 4.0 AFM, Barcelona, Spain). For each patient, the following pathological and clinical parameters were studied: disease-free survival, patient status (dead, alive), sex, age (<60 *versus* >60), histological type (squamous, large cell, adenocarcinomas) and degree of differentiation (well, moderate, poor, undifferentiated). In order to corroborate the results from the array, a second series (series 2) was analysed with a different microRNA platform and different company FEBIT, version 15.0 Geniom Biochip MPEA, Heidelberg, Germany. A third series of 119 patients (series 3) was chosen for protein study by IHC. All procedures used in the present study have been approved by the ethics committee of Hospital Vall d’Hebron.

### RNA extraction

Normal and tumour frozen tissue from the lung was used for RNA extraction from a total of 47 patients. H&E staining of the slides from frozen biopsies was performed to ensure that the tissue area would have an adequate tumour density (<60%). Total RNA was isolated with a MirVana kit (Ambion, Austin, TX, USA) according to the manufacturer’s instructions. RNA quality was assessed by Bioanalyser (RIN ratio >8). RNA from lung cancer cell lines was extracted with the same protocol from subconfluent culture dishes (ø=10 cm).

### Microarray preparation

Human microRNA microarrays (G4470B; Agilent, Santa Clara, CA, USA) containing 13 737 probes corresponding to 799 microRNAs and 22 control probes were hybridised with RNA from normal and cancer tissue of 24 NSCLC patients. Briefly, 500 ng of total RNA from each sample were chemically labelled by dephosphorylation using Calf Intestinal Alkaline Phosphatase (CIP) and ligated to Cyanine3-pCp with a T4-RNA ligase using Agilent miRNA Complete Labeling and Hyb Kit (p/n5190-0456, Agilent). Labelled samples were dried, resuspended in 18 *μ*l of nuclease-free water and treated with *in situ* hybridisation buffer for 20 h at 55 °C. Samples were then washed at room temperature for 5 min in Gene Expression Wash Buffer 1 (Agilent, Santa Clara, CA, USA) and for 5 min at 37 °C in Gene Expression Wash Buffer 2 (Agilent). The images were generated on a confocal microarray scanner G2565BA (Agilent) at 5 *μ*m resolution and quantified using Feature Extraction (Agilent).

### Real-time PCR

Quantitative real-time PCR was used to determine levels of miR-99a (Hs04231437_s1), miR-205 (ID 000509), Nanog (Hs04399610_g1), Oct3/4 (Hs04260367_gH), Sox2 (Hs01053049_s1), Snail (Hs00161904_m1), Twist (Hs01675818_s1) and housekeeping genes U6 (ID 0001093), TBP (Hs00427620_m1) and IPO8 (Hs00183533_m1) using the Assays-on-Demand Taqman Gene Expression Assays (Applied Biosystems, Foster City, CA, USA) according to the procedure previously described.^38^ qRT-PCR was performed to determine the relative RNA levels of: (1) miR-99a in 10 cancer tissues (*versus* normal) to corroborate the array data; (2) miR-99a RNA levels in H1299, H1975 and H1650 NSCLC cell lines (control and transfected with miR-99a); (3) Nanog, Oct3/4, Sox2, Snail and Twist genes in the H1299, H1975 and H1650 NSCLC cell lines expressing miR-99a *versus* control; (4) Nanog, Oct3/4, Sox2, Snail and Twist genes in H1975 mice tumours formed by miR-99a expression (*versus* control); (5) Nanog, Oct3/4, Sox2, Snail and Twist genes in 8 cancer tissues: the top 4 with highest miR-99a expression (patients 6, 26, 28 and 48 from series 1) *versus* the top 4 with the lowest miR-99a expression (patients 14, 18, 36 and 40) from the array (series 1) and (6) miR-99a in 30 cancer tissues to correlate with E2F2 and EMR2 expression. Results were conducted in triplicate in at least three independent experiments.

### Bioinformatics search

Potential miR-99a targets were predicted and analysed by using publicly available algorithm-based databases, including PicTar (http://pictar.mdc-berlin.de/), TargetScan (http://www.targetscan.org/), miRanda (http://www.microrna.org/) and DIANA-microT (http://diana.imis.athena-innovation.gr). To select the putative miR-99a mRNA targets, we focussed on those detected in more than one miRNA database. Of these, E2F2 and EMR2 were selected.

### Plasmid construction

For the miR-99a stable overexpression experiments, premiR-99a was cloned into retroviral vector miR-V that was kindly donated by Dr. R Agami (Netherlands Cancer Institute, Amsterdam, The Netherlands). Approximately 500 nt of the genomic DNA sequence that encodes for primary miR-99a and its natural flanking sequences was selected for PCR amplification, according to a previously described procedure.^39^ Primers are detailed in Supplementary Table 7.

For performing luciferase assay, cloning to pmirGLO pDNA (Promega Corporation, Madison, WI, USA) containing the luciferase reporter and *Renilla* gene has been performed with 1088 bp 3′-UTR-E2F2 mRNA sequence (NCBI Reference Sequence: NM_004091.3) artificially synthesised (Thermo Fisher Scientific, Waltham, MA, USA) and 1024 bp 3′-UTR-EMR2 (ADGRE2) (NCBI Reference Sequence: NM_013447.3) artificially synthesised (ThermoFisher). For the mutant E2F2 miR-99 construct, the seeding sequence was replaced with 5′-GGGAGATATGAATGGTACcaaTG-3′ having recognition site for *Kpn*I restriction enzyme (Figure 3a and Supplementary Table 8). For the mutant EMR2 miR-99 construct the seeding sequence was replaced with 5′-GTTGTTCTCTAGTTCTAaGcttTT-3′ having recognition site for *Hin*dIII restriction enzyme (Figure 3a and Supplementary Table 8). All the subcloned inserts had *Nhe*I (3′-GCTAGC-5′) and *Sbf*I (3′-CCTGCAGG-5′) restriction sites. In all cases, the cloned PCR products were validated by sequencing (data not shown).

### Protein analysis

Total cell lysates were prepared from a subconfluent 10 cm dish. Cells were washed in PBS and lysed in 1 ml of lysis buffer (50 mM Tris-HCl, pH 7.5, 1% NP-40, 10% glycerol, 150 mM NaCl, plus 2 mM complete protease inhibitor cocktail) (Roche Diagnostics, Barcelona, Spain). From each sample, 50 *μ*g of protein, quantified with the Bio-Rad protein assay (Bio-Rad, Hercules, CA, USA), was analysed by gel electrophoresis on a 6–12% SDS polyacrylamide gel and transferred onto a nitrocellulose membrane. The following antibodies were used for western blot analysis: E2F2 (sc632, Santa Cruz, Heidelberg, Germany), EMR2 (sc34334, Santa Cruz), E-cadherin (3195, Cell Signaling Technology Europe Leiden, The Netherlands), N-cadherin (ab18203, Abcam, Cambridge, UK) and *β*-actin (A2228, Sigma-Aldrich Quimica SL, Madrid, Spain). In all cases, membranes were incubated overnight at 4 °C with the primary antibodies in T-TBS with 5% nonfat dry milk. The membranes were then washed with T-TBS and incubated with horseradish peroxidase-conjugated anti-mouse or anti-rabbit secondary antibody (Sigma-Aldrich). After additional washes with T-TBS, the antigen–antibody complexes were visualised with an enhanced chemiluminescence kit (Merck Chemicals & Life Science, Madrid, Spain).

### Cell culture

H1975, H1299 and H1650 NSCLC cell lines were obtained from the American Type Culture Collection (ATCC, Manassas, VA, USA). The three cell lines were grown in RPMI-1640 medium (Lonza Bioscience Sarl, Bruguières, Francia). Media were supplemented with 10% FBS (Sigma-Aldrich Quimica SL), 100 U/ml penicillin and 100 *μ*g/ml streptomycin. All of the cells were grown at 37 °C in a humidified incubator with 5% CO_2_. Cells were passaged regularly twice per week and maintained at subconfluence.

### Transfection–transduction

Transient transfection of H1299, H1650 and H975 cells using Lipofectamine 2000 (Thermo Fisher Scientific, Waltham, MA, USA) or jetPEI (Polyplus) was performed with the synthetic precursors of miR-99a called pre-miR-99a or miR-99a mimic (designated here as miR-99a) (ID: AM17100; (Thermo Fisher Scientific)), anti-miR-99a (ID: 10719; Thermo Fisher Scientific), a Cy3 dye-labelled negative control (ID: AM17020; Thermo Fisher Scientific) or negative control miR-C (ID: AM17110; Thermo Fisher Scientific). H1299, H1650 and H1975 cells were seeded at 2.5 × 10^5^ and 2.0 × 10^5^ and 1.5 cells × 10^5^ per well, respectively, in 6-well plates and transiently transfected with miR-99a or anti-miR-99a to a final concentration of 80 nM with Lipofectamine according to the manufacturer’s instructions. At 72 h after transfection, cells were counted and the cellular lysates were collected for analysis of the protein expression of the selected putative miR-99a targets. Transfection efficiency was determined by fluorescence microscopy and compared with Cy3 dye-labelled negative control.

To study the transient effects of EMR2 or E2F2 silencing, H1299, H1650 and H1975 cells were seeded at 2 × 10^5^ cells per well in a 6-well plate and transfected the following day with the indicated siRNAs or controls (NT-siRNA or scramble -Sc-) using Lipofectamine. After 48 h, cells were counted and lysed for RNA and protein extraction as previously described.^40^

For the stable expression, miR-99a (cloned in miR-V, designated miRV-99a), miR-V and miRV-GFP were included, the latter for checking infection efficiency that in all cases was ~100%. Then, 30 *μ*g of each retroviral vector was transfected into Phoenix cells in 10 cm culture plates with FuGENE (Roche Diagnostics, Barcelona, Spain). The viral particles in the supernatant were harvested 48 h after transfection and used to infect H1299, H1650 and H1975 cells. Each stably transduced cell line was selected with blasticidin (10 *μ*g/ml) for 12 days. In all cases, parental cells (uninfected) were maintained and treated with blasticidin for checking appropriate selection. Results were conducted in triplicate in at least three independent experiments.

For the preparation of the analysis of the SP cells, H1299 and H1975 cells were transduced using Lipofectamine 2000 concomitantly with Cy3 dye-labelled negative control plus miR-99a *versus* miR-C (1 : 10 ratio) in order to select only transfected cells.

### Growth curves

H1975, H1299 and H1650 cells were seeded at 1 × 10^6^ cells per 10-cm plate. Parental cells for each cell line, the control cells (miRV-GFP-infected), and cells that expressed miR-99a (miRV-99a) were grown simultaneously. Every 3 days, the cells from each cell line were counted and reseeded at a density of 1 × 10^6^ cells per 10 cm plate, as indicated by the 3T3 protocol described.^39^ In addition, 5 × 10^4^ cells per well were reseeded every 3 days in 24-well plates in triplicate and fixed. Cell staining was performed with crystal violet. For CDDP (Sigma-Aldrich) treatment, H1975 cells were tritated at different concentrations and 20 *μ*M were chosen. Cells (1 × 10^3^) were seeded in 96 well-plates by quintuplicate and treated the next day with CDDP. Cells were fixed and measured for viability with the MTS assay after 48 h of treatment. Results were conducted in triplicate in at least three independent experiments.

### Luciferase reporter assay

The luciferase experiments were performed in HEK293T cells. HEK293T cells were seeded at 1 × 10^4^ cells per well in a 96-well plate and were transfected the following day with Lipofectamine with the following molecules: the synthetic miRNA precursor miR-99a (ID: AM17100; Thermo Fisher Scientific), and negative control miR-C (ID: AM17110; Ambion), Cy3 (ID: AM17020; Thermo Fisher Scientific) and the pmirGLO plasmid (Promega Corporation, Madison, WI, USA) containing the luciferase reporter and also the *Renilla* gene (control) *versus* pmirGLO3′-UTR-E2F2 or pmirGLO3′-UTR-EMR2. The transfection efficiency was ∼95%, and luciferase activity was measured 48 h after transfection with the dual luciferase reporter assay as previously described.^39^ In each case, the miR-99a concentrations were measured by titrating the miR-99a with each pmirGLO3′-UTR mRNA construct to establish a dose–response relationship between 10 and 80 nM (data not shown). Results were conducted in triplicate in at least three independent experiments.

### Cell cycle analysis

For the cell cycle analysis, a fluorescence-activated cell sorting Calibur flow cytometer (FACS Calibur, Becton Dickinson, E0772; BD Biosciences, San Jose, CA, USA) was used to analyse H1299, H1650 and H1975 cells that transiently or stably expressed miR-99a *versus* negative control. One million cells from each sample were fixed in 70% ethanol for 15 min at −20 °C, treated with 100 *μ*g/ml RNase A (Sigma-Aldrich) and stained with 5 *μ*g/ml of propidium iodide (PI) (Sigma-Aldrich). For each sample, 2 × 10^4^ cells were analysed, and the percentage of cells in each phase of the cell cycle was calculated based on the DNA content determined with FACS Express Software. Results were conducted in triplicate in at least three independent experiments.

### Annexin V–APC apoptosis analysis

H1299, H1650 and H1975 cells were transfected with miR-99a *versus* control and infected with miRV-99a or miRV-GFP and were selected with blasticidin for stable expression. The quantification of apoptotic cells was performed with the Annexin V-APC Detection Kit (Thermo Fisher Scientific) according to the manufacturer’s instructions. The samples were then analysed by FACS. For each sample, 2 × 10^4^ cells were analysed and the results were analysed with the FACS Express Software. The results were confirmed in at least three independent experiments.

### Animal model

Mouse from NMRI-FOXn1 nu/nu strain (Janvier Labs, Saint Berthevin Cedex, France) were used for xeno-injection. H1975 lung cancer cells (1 × 10^6^ cells) were transiently transfected with 100 nM of control microRNA (miR-C, non-silencing control) or miR-99a mimic. After 36 h, viable cells were harvested and injected subcutaneously (1 × 10^6^/mouse) in the flank of 6-week-old female NMRI-FOXn1 nu/nu in 300 *μ*l of PBS and matrigel (1 : 1). H1975 cells were injected into a total of 16 mice (8 mice were xeno-injected with control microRNA and 8 mice with miR-99a). Tumour volume was measured every 2–3 days during 2 weeks using an electronic calliper. At the respective scheduled surgery, mice were killed and tumours removed and weighted. Tumours were then fixed in 10% formalin, paraffin-embedded, and 5 *μ*m sections were H&E stained. A portion of each tumour was snap-frozen and reserved for RNA and protein studies.

### Analysis of SP cells

SP analysis was performed as described with slight modifications.^41^ Briefly, NSCLC cells were digested with TrypLE Express (Thermo Fisher Scientific), washed with PBS and resuspended at a density of 1 × 10^6^ cells/ml in prewarmed RPMI-1640 culture medium (Thermo Fisher Scientific, Waltham, MA USA) supplemented with 2% FBS and 10 mM 4-(2-hydroxyethyl)-1-piperazineethanesulfonic acid (HEPES) (Sigma-Aldrich). Then, the cells were incubated at 37 °C for 120 min with 5 *μ*g/ml Hoechst 33342 dye (Thermo Scientific). Control cells were incubated with 50 *μ*M verapamil (Sigma-Aldrich) for 15 min at 37 °C before the addition of Hoechst dye to validate the SP detection (data not shown). For cell dead discrimination, PI (Sigma-Aldrich) at 5 *μ*g/ml was added to the cells before FACS analysis. Cell samples were analysed and sorted using FACSAria II flow cytometer (BD Biosciences).

### Immunohistochemistry

The expressions of E2F2 (sc-632, Santa Cruz), EMR2 (sc-34334, Santa Cruz), *β*-catenin (9562, Cell Signaling) and vimentin (V6389, Sigma-Aldrich) proteins were studied by IHC in the 119 patient samples (series 3) with lung cancer. *β*-Catenin was located at the cell membrane, E2F2 and vimentin at cytoplasmic level, and EMR2 nuclear as previously described.^27^ Paraffin-embedded biopsies were included in tissue microarrays (TMAs) as previously described.^39^ The sections were incubated at RT during 2 h with the indicated antibodies and the immunostaining was performed using the ChemMate DAKO EnVision Detection Peroxidase/DAB kit K4065 (DAKO Diagnóstics, Barcelona, Spain). Quantification of the reaction was performed using the histoscore system as previously described.^39^

### Statistical analysis

All sample data were analysed using SPSS software (version 11.0, SPSS, IBM SPSS, Madrid, España), and *P*<0.05 was considered statistically significant (*P<0.05; **P<0.01). To identify differentially regulated microRNAs, moderated paired *t*-tests were applied using limma.^42^ Pairwise differences between groups were analysed using Student’s *t*-test (proliferation curves, spheroids formation, RNA study by qRT-PCR, protein staining by IHC, cell cycle profiles, apoptosis, migration, invasion and adhesion). All data described in this manuscript will be shared with the scientific community upon request.

## Publisher’s Note

Springer Nature remains neutral with regard to jurisdictional claims in published maps and institutional affiliations.
