# Supplementary material for: miR-99a reveals two novel oncogenic proteins E2F2 and EMR2 and represses stemness in lung cancer
Source: Cell Death Dis. 2017 Oct 26;8(10):e3141–. doi: 10.1038/cddis.2017.544 (PMC5680913; doi:10.1038/cddis.2017.544)
Supplement: Supplementary Figure legends [file cddis2017544x1.doc]

**Supplementary Figure legends**

**Fig. S1.** microRNA signature distinguishes healthy from tumour tissue in NSCLC patients. (*A*) Ninety-nine microRNAs showed a distinct behaviour in normal versus tumour tissue (supplementary Table 2). Based upon the differential expression of 97 microRNAs, a diagram clustering the samples is shown. (*B*) Correlation-based clustering using 97 differentially regulated microRNAs in correlation with the histological type of tumours. The ratio of tumour/normal microRNA expression was considered. A significant association between the microRNA-based 3 clusters and tumour types was found (*p*= 0.01). T1= adenocarcinomas, T2= large cell carcinomas and T3= squamous cell carcinomas. (*C*) Correlation-based clustering using 97 differentially regulated microRNAs in correlation with the differentiation of the tumours (*D*) The ratio of tumour/normal microRNA expression was considered. A significant association between the microRNA-based 3 clusters and differentiation degree was found (*p*=0.04). D1= well differentiated tumours, D2= moderately differentiated tumours, D3= poorly differentiated tumours and D4= undifferentiated tumours.

**Fig. S2.** Correlation of the expression of microRNAs with pathological characteristics. (*A*) Correlation of the expression of microRNA, miR-205, in the tumour tissue with the specific histologic type of squamous cell carcinoma (*p*=0.02). T1= adenocarcinomas, T2= large cell carcinomas and T3= squamous cell carcinomas. (*B*) Correlation of the expression of microRNAs, miR-101 (*p*=0.04), miR-101* (*p*=0.04), miR-181a* (*p*=0.03), miR-30b (*p*=0.03) and miR-338-3p (*p*=0.01), with the degree of differentiation of the tumours. D1= well-differentiated tumours, D2= moderately differentiated tumours, D3= poorly differentiated tumours and D4= undifferentiated tumours.

**Fig. S3.** Relative mRNA levels. (*A*) Example of representative mRNA levels of 10 lung cancer biopsies by qRT-PCR (Series 1, Platform 1). (*B*) Correlation of the results from both techniques by qRT-PCT and microarray platform for the samples analysed in S3A.

**Fig. S4.** miR-99a decreases proliferation. (*A*) Example of morphological changes of H1975 cells transiently expressing miR-99a versus control. (*B*) Example of morphological changes in lung cancer cell lines stably expressing miRV-99a versus control (empty vector miR-V). (C) Relative RNA expression of miR-99a in the indicated cells stably expressing miRV-99a versus control (empty vector miR-V). Infected cells were blasticidin-selected for 12 days and then analysed for miR-99a levels. (*D*) Cell number of miRV-99a expressing cells after 12 days of selection with blasticidin versus controls. (*E*) Number of death cells (counted by trypan blue exclusion) in relation to 100 living cells.

**Fig. S5**. Result of stable expression of miR-99a on cell proliferation. The indicated cell lines were infected with 1:3 and 1:10 viral dilutions for stable expression of miR-99a. Note that even at 1:10 dilution, miR-99a suppresses cell proliferation.

**Fig. S6**. Impact of different viral dilutions on H1299 cells. (*A*) miR-99a levels in stably expressing miR-99a H1299 cells versus controls (1:3 and 1:10 dilutions are shown; undiluted virus is shown on Fig. 2A). (*B*) Apoptotic events in H1299 cells infected with the indicated viral dilutions and further selected with blasticidin for 10 days.

**Fig. S7**. EMR2 and E2F2 protein expression in lung cancer cell lines. (*A*) Western Blot densitometric measurements of the EMR2 and E2F2 proteins upon transduction with miR-99a vs. control in the indicated cell lines. (*B*) Western Blot densitometric measurements of the E2F2 and EMR2 proteins upon transduction with anti-miR-99a. (*C*) Representative Western-blots of EMR2 (upper panel) and E2F2 (lower panel) proteins detected in lung cancer cells after 48 h transductionwith the indicated siRNAs versus control (scramble, Sc) siRNA.

**Fig. S8.** Potential EMT indicators. Relative invasion (*A*) and adhesion (*B*) of H1299, H1650 and H1975 cells transduced with miR-99a, anti-miR-99a or control. Relative invasion (*C*) and adhesion (*D*) of H1299, H1650 and H1975 cells transduced with scrambled (SC) control, siRNA E2F2 or siRNA EMR2.

**Fig. S9**. Representative pictures of migration and invasion assays summarised in Fig. S8. (*A*) Representative images of migrated H1299, H1650 and H1975 cells after transduction with miR-99a and anti-miR. (*B*) Invasion picture of miR-99a expressing H1299 and H1975 cells. (*C*) Representative images of migrated H1299, H1650 and H1975 cells after transduction with the siRNA-E2F2 and siRNA-EMR2.

**Fig. S10.** Markers of EMT and stemness. (*A*) Representative Western blots showing E-Cadherin and N-Cadherin protein expression in H1299, H1650 and H1975 transduced cells after transfection with corresponding miRNAs for 48 h (left panel). Densitometric quantification of the protein expression levels (right panel). (*B*) Relative mRNA levels of the indicated genes in the spheroid CSC-like cells from generation 1 (G1) to generation 3 (G3) in non-adherant conditions vs. adhearant (parental) cells.

**Fig. S11.** SP discrimination assay. H1975 (*A*) and H1299 (*B*) cells transduced with the siRNA-E2F2, siRNA-EMR2 or control (SC) were analysed for the percentage of the SP cells. Note that each siRNA decreases the number of SP cells by more than half. Results are representative of three independent experiments. (*C*) Relative qRT-PCR of the whole level of miR-99a in the whole bulk of tumours from patients studied in Fig. 6H. Np, pool of normal tissues. (*D*) Relative qRT-PCR of miR-99a levels from the mice tumors formed by H1975 cells transduced with miR-99a and control (miR-C).

**Tables**

**Table S4**. Total of 95 deregulated microRNAs found in the second series of patients. Twenty-nine microRNAs were commonly deregulated between the first and second series of patients (Fig. 1A), while deregulation of 44 microRNAs were not confirmed. In orange are indicated 22 newly found deregulated microRNAs (their analysis were not included in the first series).
