# Supplementary material for: miR-99a reveals two novel oncogenic proteins E2F2 and EMR2 and represses stemness in lung cancer
Source: Cell Death Dis. 2017 Oct 26;8(10):e3141–. doi: 10.1038/cddis.2017.544 (PMC5680913; doi:10.1038/cddis.2017.544)
Supplement: Supplementary Figures [file cddis2017544x2.pdf]

Supplementary Figure 1

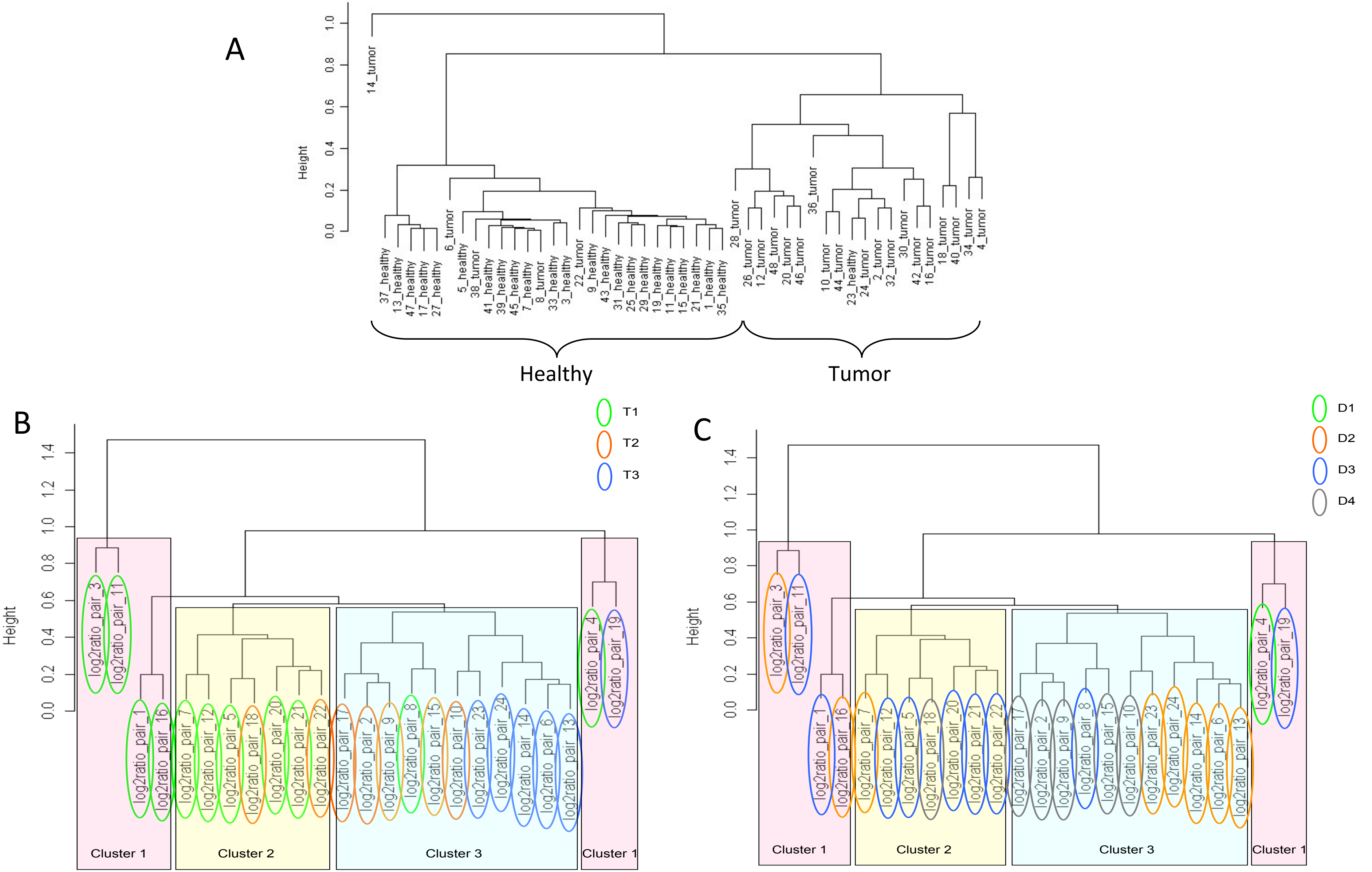

Supplementary Figure 2

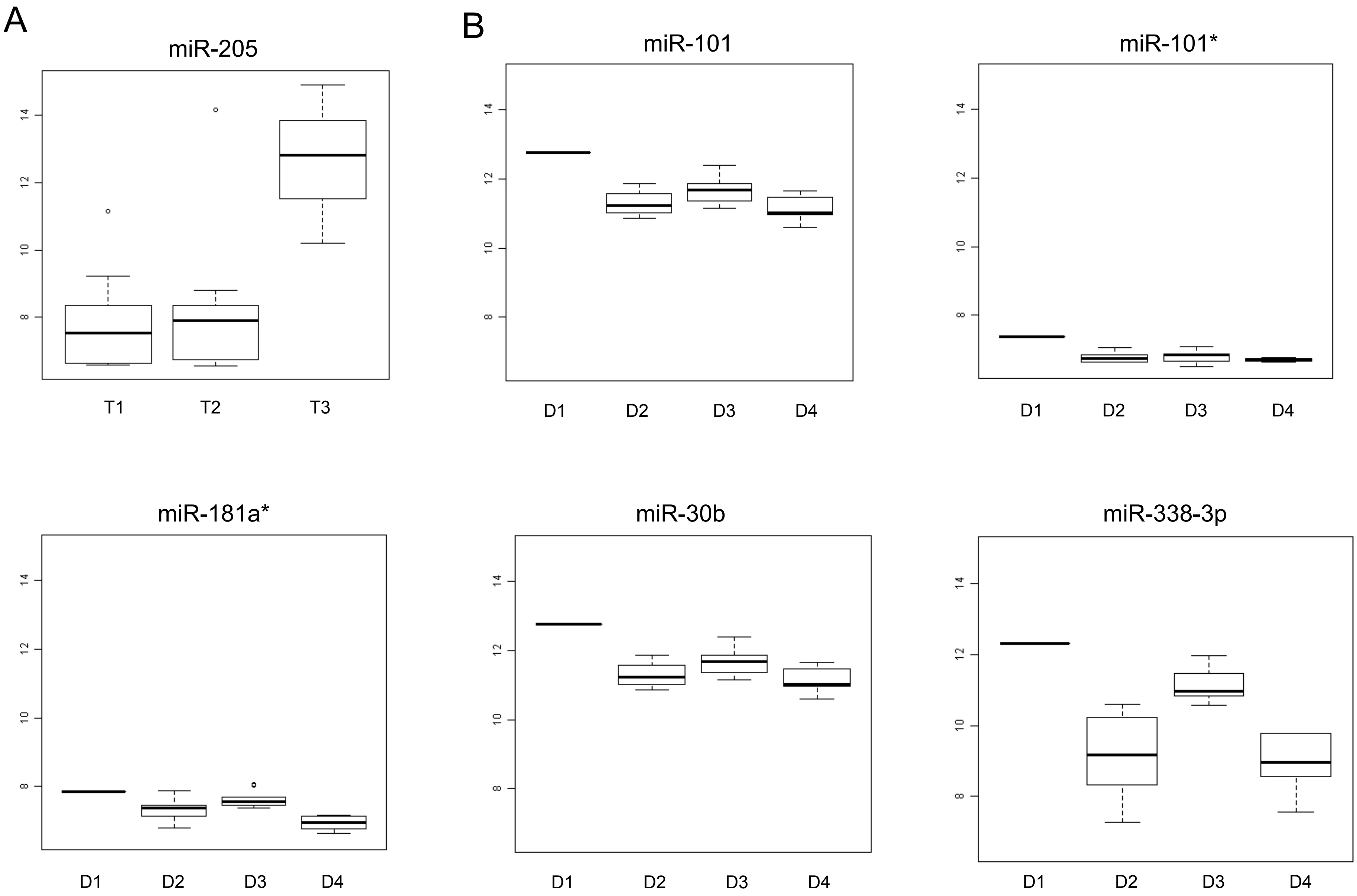

A

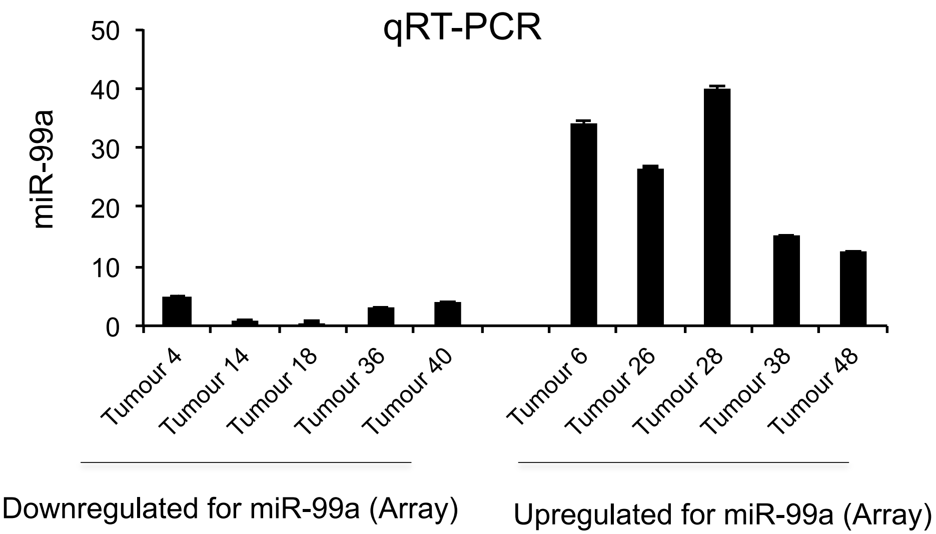

B

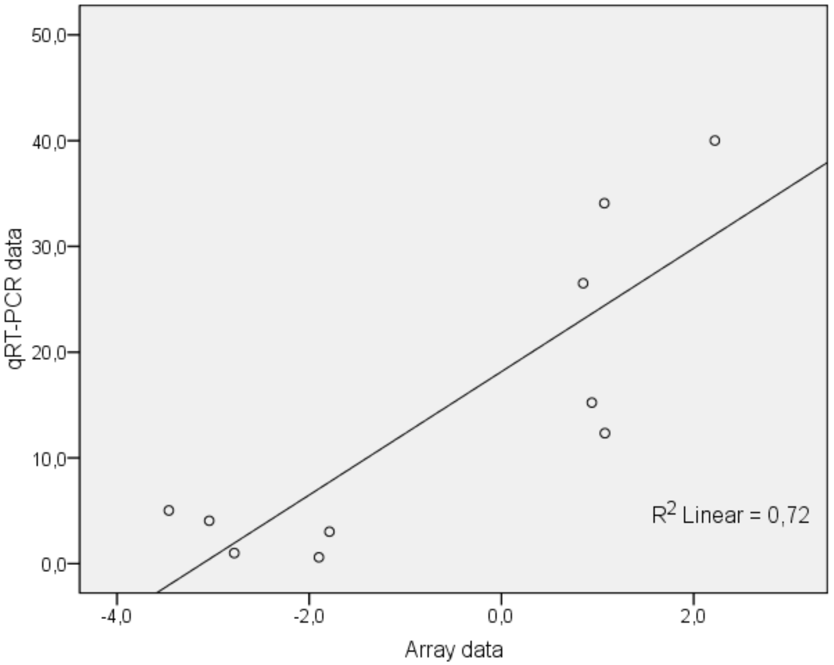

Supplementary Figure 3

Supplementary Figure 4

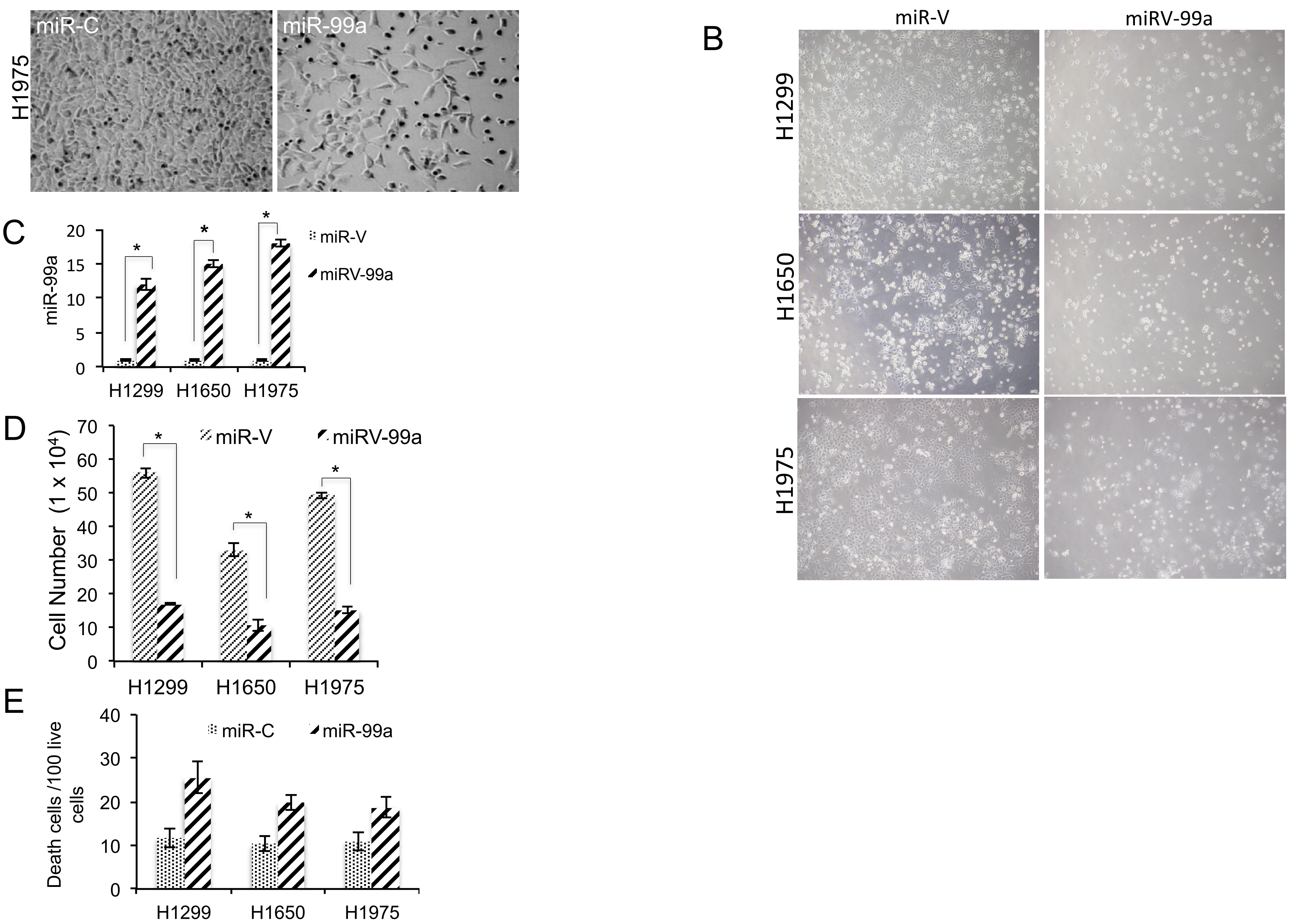

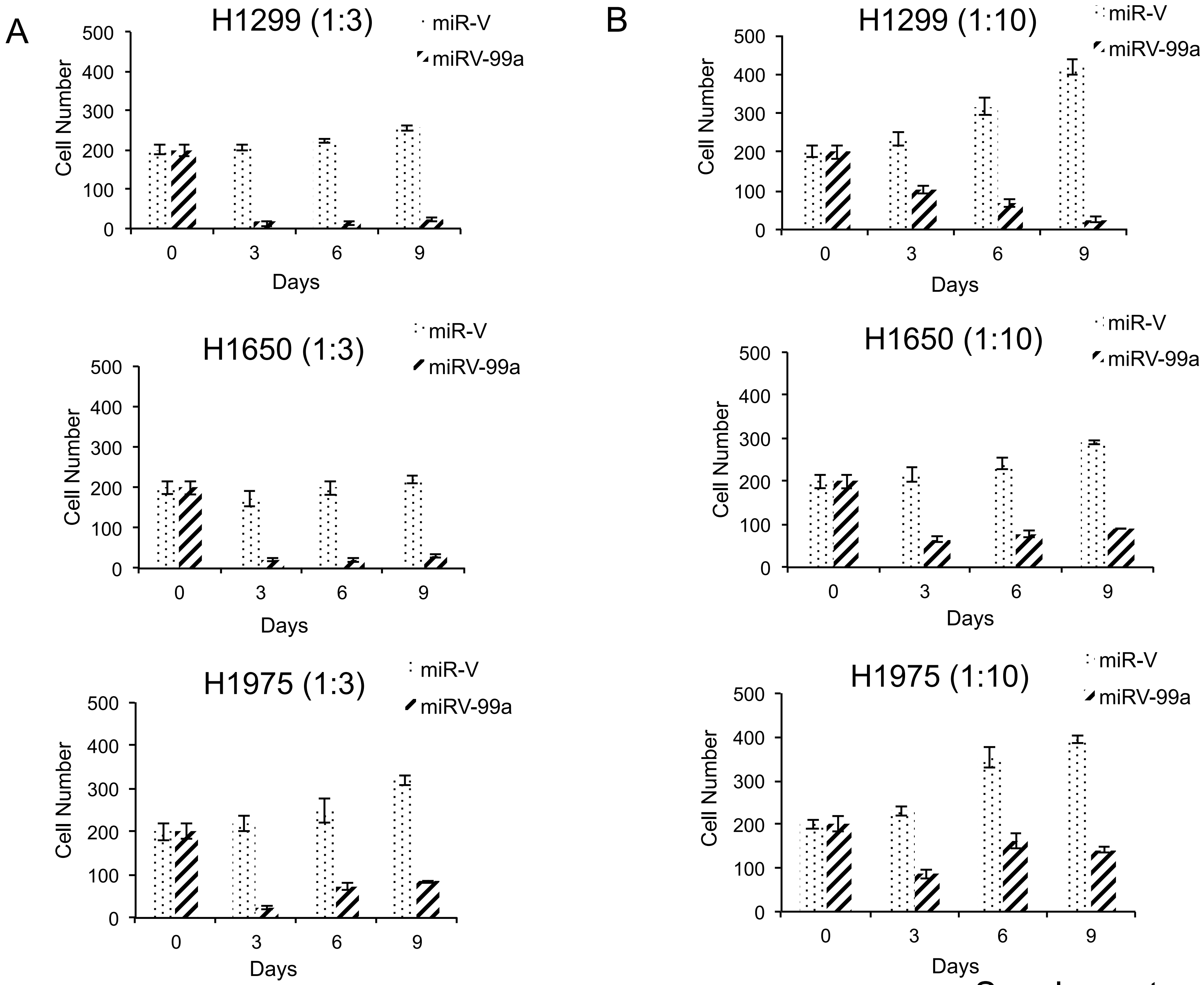

Supplementary Figure 5

Supplementary Figure 6

A

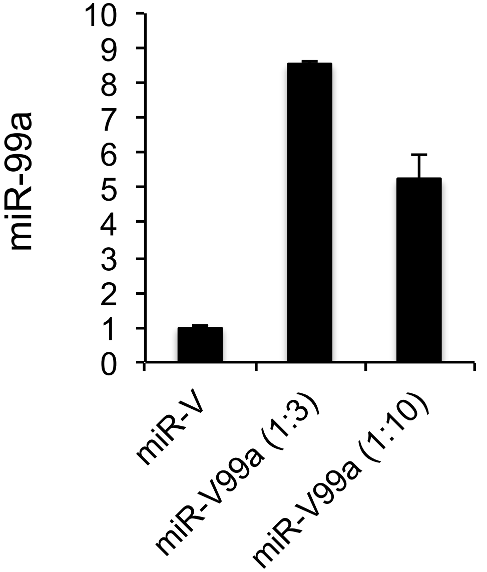

B

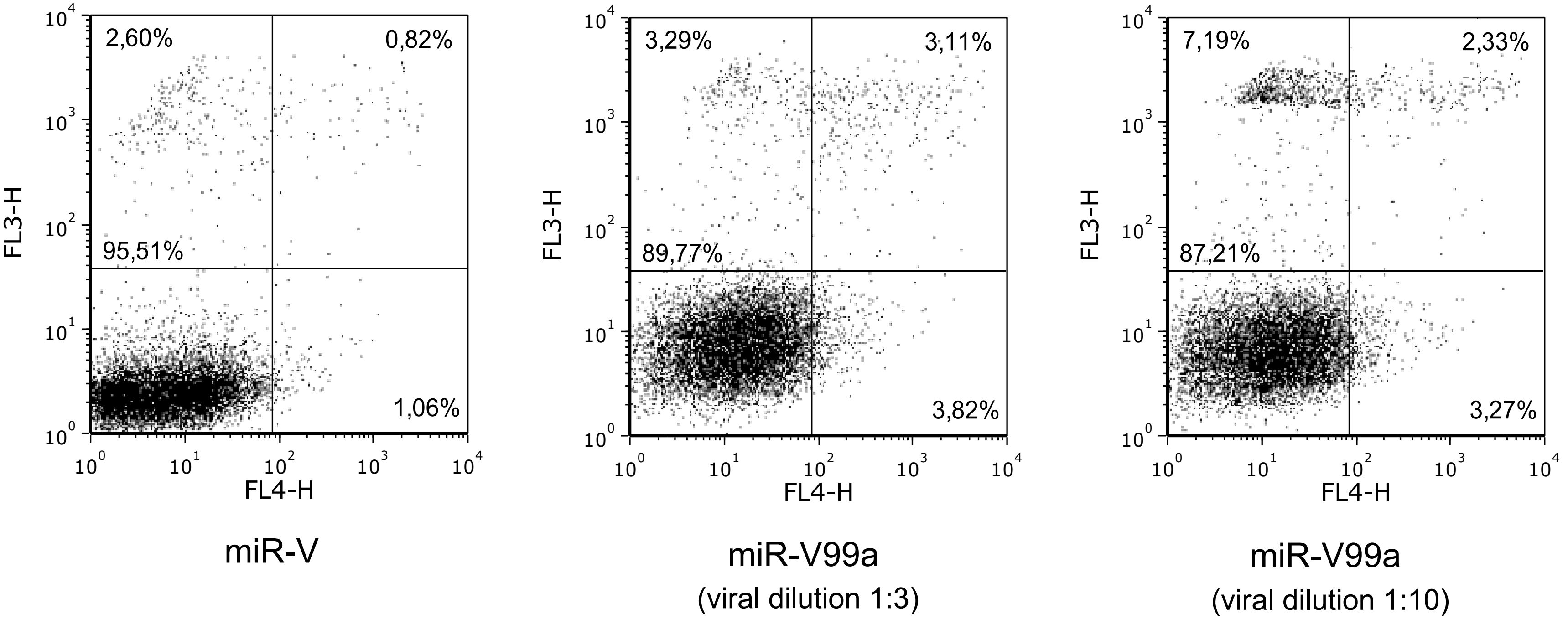

Supplementary Figure 7

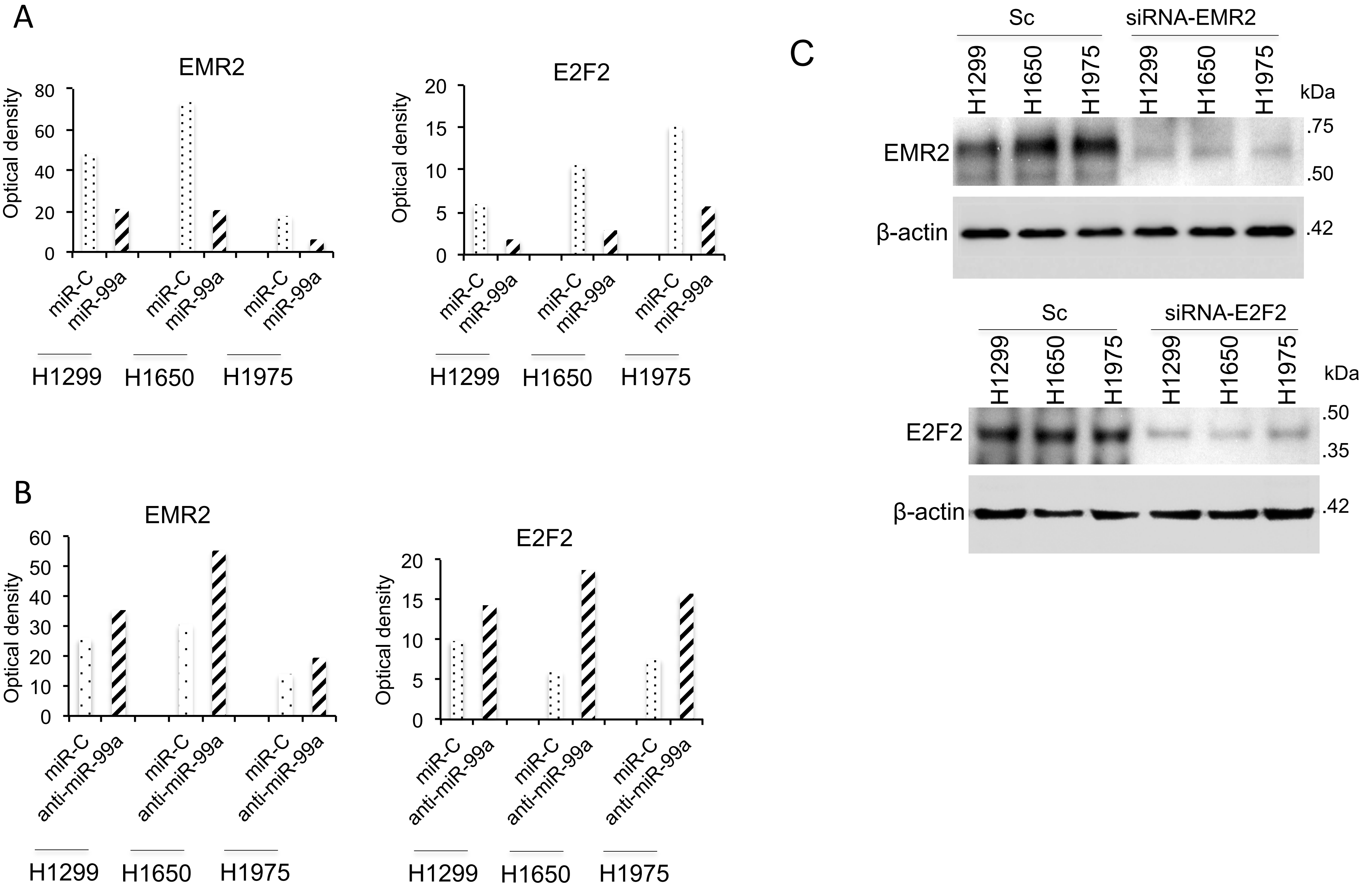

Supplementary Figure 8

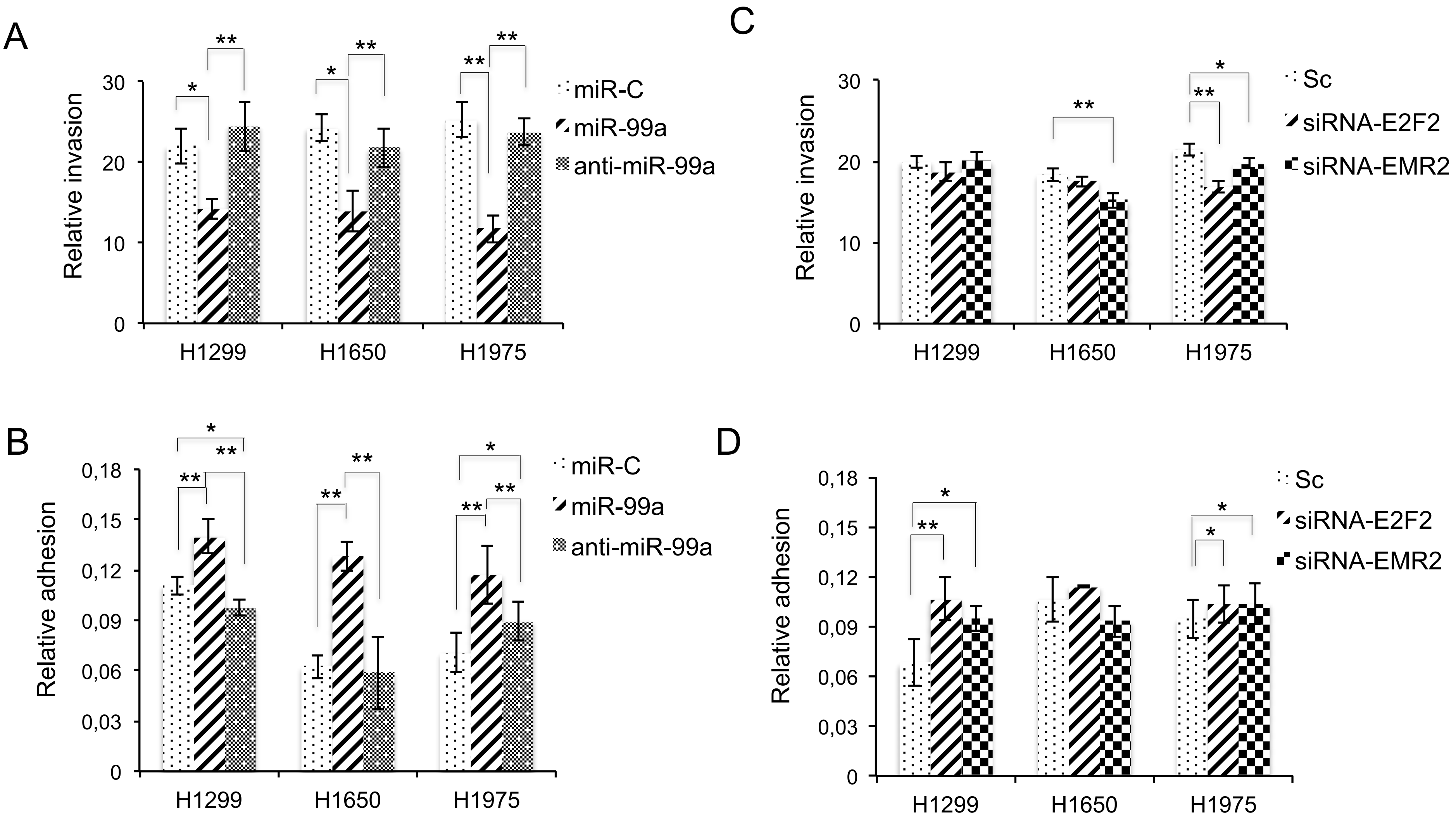

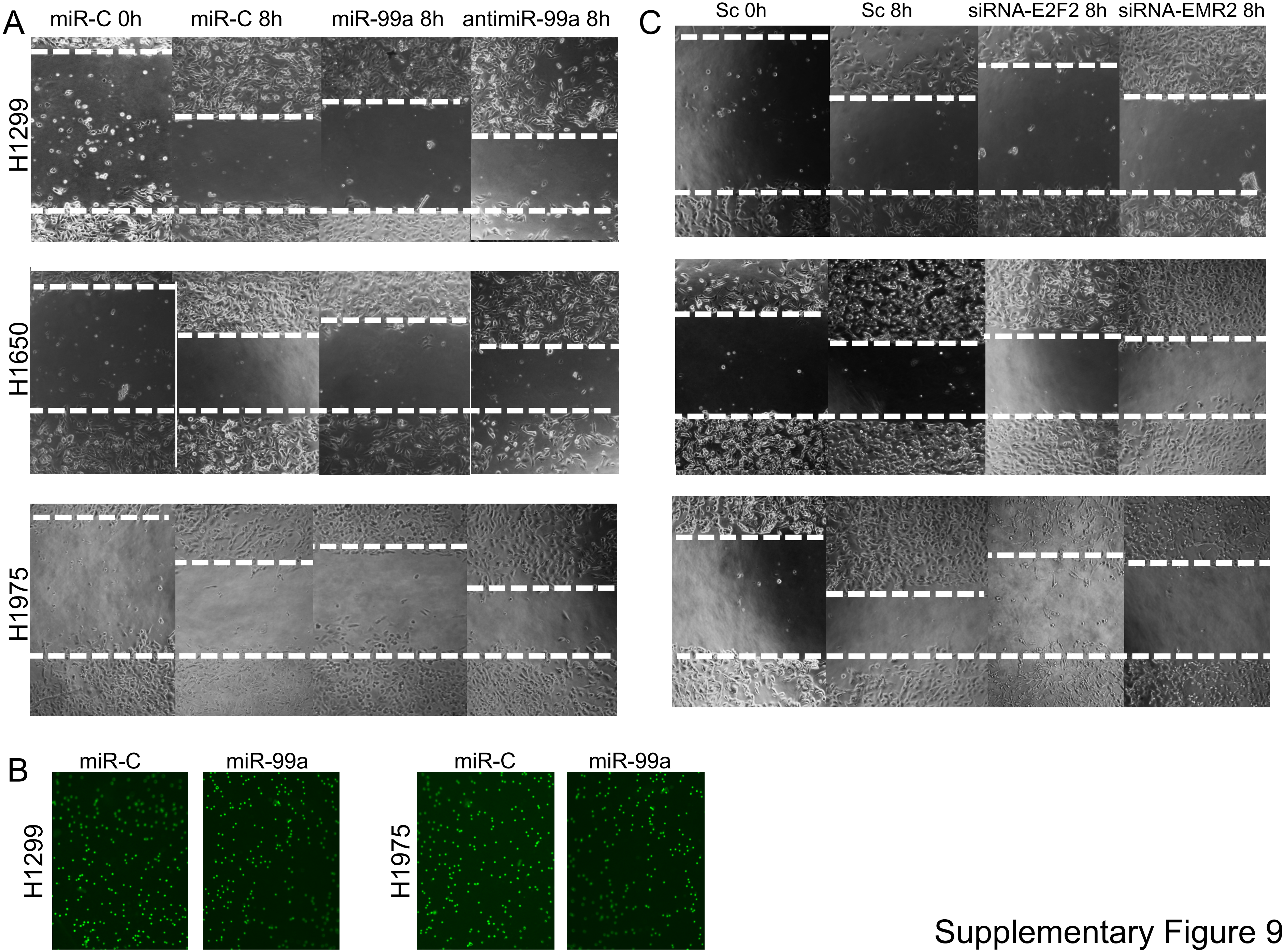

Supplementary Figure 9

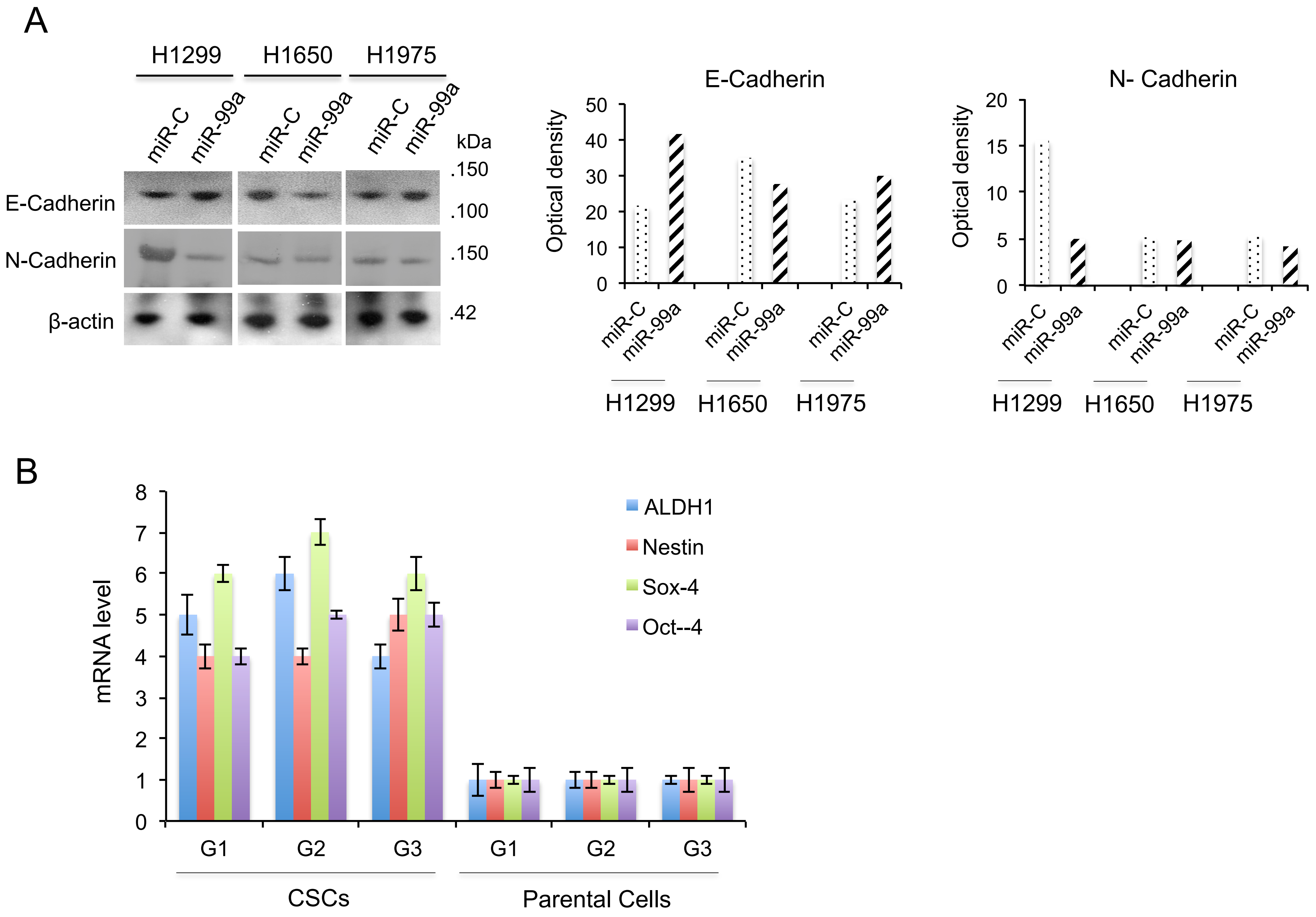

Supplementary Figure 10

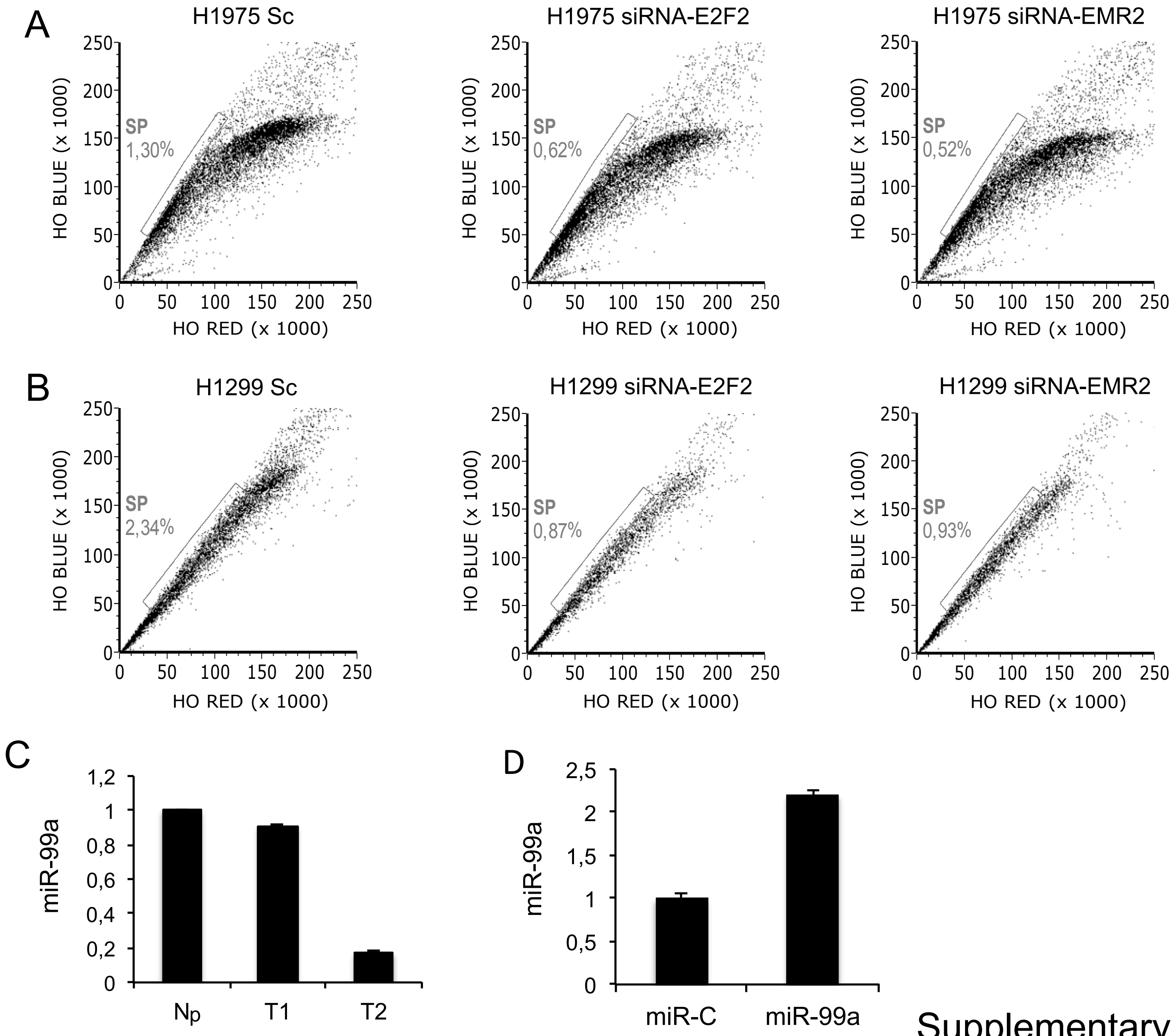

Supplementary Figure 11
