# Supplementary material for: miR-99a reveals two novel oncogenic proteins E2F2 and EMR2 and represses stemness in lung cancer
Source: Cell Death Dis. 2017 Oct 26;8(10):e3141–. doi: 10.1038/cddis.2017.544 (PMC5680913; doi:10.1038/cddis.2017.544)
Supplement: Supplementary Table 2 [file cddis2017544x4.doc]

**Supplementary Table 2**. Differentially expressed microRNAs when comparing tumour versus normal tissue of 24 NSCLC patients.

| microRNA | Fold-change ^b^ | Adjusted *P* value | Strongest regulation |
| --- | --- | --- | --- |
| hsa-miR-205 | 4,01 | 0,01 | squamous |
| hsa-miR-96 | 3,23 | 0,00 | large cell |
| hsa-miR-210 | 2,77 | 0,00 | large cell |
| hsa-miR-141 | 2,76 | 0,00 | large cell |
| hsa-miR-494 | 2,45 | 0,00 | large cell |
| hsa-miR-200b | 2,38 | 0,00 | large cell |
| hsa-miR-130b | 2,33 | 0,00 | large cell |
| hsa-miR-7 | 2,17 | 0,00 | large cell |
| hsa-miR-31 | 2,16 | 0,01 | large cell |
| hsa-miR-200c | 2,13 | 0,00 | large cell |
| hsa-miR-429 | 2,03 | 0,01 | large cell |
| hsa-miR-9* | 1,94 | 0,00 | large cell |
| hsa-miR-21 | 1,85 | 0,00 | squamous |
| hsa-miR-31* | 1,83 | 0,01 | large cell |
| hsa-miR-196a | 1,78 | 0,00 | large cell |
| hsa-miR-196b | 1,77 | 0,00 | large cell |
| hsa-miR-135b | 1,73 | 0,05 | large cell |
| hsa-miR-21* | 1,70 | 0,01 | adenocarcinoma |
| hsa-miR-106b | 1,70 | 0,00 | large cell |
| hsa-miR-19a | 1,70 | 0,00 | large cell |
| hsa-miR-183 | 1,64 | 0,00 | large cell |
| hsa-miR-19b | 1,53 | 0,00 | large cell |
| hsa-miR-93 | 1,50 | 0,01 | large cell |
| hsa-miR-141* | 1,49 | 0,00 | large cell |
| hsa-miR-18a | 1,48 | 0,02 | large cell |
| hsa-miR-9 | 1,43 | 0,00 | large cell |
| hsa-miR-17 | 1,43 | 0,05 | large cell |
| hsa-miR-425 | 1,41 | 0,00 | large cell |
| hsa-miR-193b | 1,41 | 0,00 | squamous |
| hsa-miR-182 | 1,38 | 0,00 | large cell |
| hsa-miR-574-5p | 1,38 | 0,01 | large cell |
| hsa-miR-200a* | 1,35 | 0,00 | large cell |
| hsa-miR-17* | 1,35 | 0,02 | large cell |
| hsa-miR-151-3p | 1,33 | 0,04 | large cell |
| hsa-let-7i | 1,33 | 0,01 | squamous |
| hsa-miR-301b | 1,31 | 0,00 | large cell |
| hsa-miR-149 | 1,28 | 0,00 | squamous |
| hsa-miR-200b* | 1,21 | 0,02 | large cell |
| hsa-miR-101* | -1,20 | 0,00 | large cell |
| hsa-miR-1224-5p | -1,22 | 0,01 | adenocarcinoma |
| hsa-miR-671-5p | -1,22 | 0,03 | adenocarcinoma |
| hsa-miR-454 | -1,22 | 0,01 | adenocarcinoma |
| hsa-miR-23ª | -1,23 | 0,01 | adenocarcinoma |
| hsa-miR-143* | -1,24 | 0,00 | large cell |
| hsa-miR-181b | -1,26 | 0,01 | large cell |
| hsa-miR-186 | -1,26 | 0,01 | large cell |
| hsa-let-7d | -1,27 | 0,04 | adenocarcinoma |
| hsa-miR-187* | -1,27 | 0,00 | squamous |
| hsa-miR-26a | -1,28 | 0,01 | large cell |
| hsa-miR-181c | -1,30 | 0,03 | squamous |
| hsa-miR-150* | -1,30 | 0,03 | adenocarcinoma |
| hsa-miR-181a* | -1,30 | 0,00 | large cell |
| hsa-miR-125a-5p | -1,30 | 0,00 | large cell |
| hsa-miR-30c-2* | -1,31 | 0,00 | squamous |
| hsa-miR-29c* | -1,34 | 0,01 | squamous |
| hsa-miR-582-5p | -1,35 | 0,02 | squamous |
| hsa-miR-16 | -1,36 | 0,02 | adenocarcinoma |
| hsa-miR-652 | -1,37 | 0,00 | squamous |
| hsa-miR-30e* | -1,37 | 0,00 | large cell |
| hsa-let-7b | -1,38 | 0,00 | large cell |
| hsa-miR-134 | -1,40 | 0,02 | squamous |
| hsa-miR-101 | -1,40 | 0,00 | large cell |
| hsa-let-7ª | -1,41 | 0,00 | squamous |
| hsa-miR-455-3p | -1,46 | 0,00 | large cell |
| hsa-miR-26b | -1,48 | 0,02 | large cell |
| hsa-miR-145* | -1,48 | 0,00 | large cell |
| hsa-let-7c | -1,49 | 0,01 | large cell |
| hsa-miR-133b | -1,50 | 0,00 | large cell |
| hsa-miR-139-5p | -1,51 | 0,00 | squamous |
| hsa-miR-125b | -1,53 | 0,02 | large cell |
| hsa-miR-181a | -1,59 | 0,00 | large cell |
| hsa-miR-572 | -1,60 | 0,00 | squamous |
| hsa-miR-199b-5p | -1,67 | 0,05 | large cell |
| hsa-miR-140-5p | -1,75 | 0,00 | large cell |
| hsa-miR-30d | -1,76 | 0,00 | squamous |
| hsa-miR-130a | -1,78 | 0,00 | large cell |
| hsa-miR-140-3p | -1,79 | 0,00 | large cell |
| hsa-miR-30c | -1,85 | 0,00 | large cell |
| hsa-miR-223 | -1,88 | 0,04 | squamous |
| hsa-miR-143 | -1,91 | 0,00 | large cell |
| hsa-miR-1 | -1,93 | 0,00 | large cell |
| hsa-miR-99a | -2,00 | 0,01 | large cell |
| hsa-miR-30b | -2,09 | 0,00 | squamous |
| hsa-miR-126* | -2,10 | 0,00 | large cell |
| hsa-miR-638 | -2,18 | 0,00 | squamous |
| hsa-miR-497 | -2,38 | 0,00 | large cell |
| hsa-miR-144* | -2,40 | 0,00 | large cell |
| hsa-miR-486-5p | -2,41 | 0,00 | large cell |
| hsa-miR-195 | -2,41 | 0,00 | large cell |
| hsa-miR-145 | -2,53 | 0,00 | large cell |
| hsa-miR-551b | -2,62 | 0,00 | large cell |
| hsa-miR-218 | -2,77 | 0,00 | large cell |
| hsa-miR-30a* | -3,00 | 0,00 | squamous |
| hsa-miR-30a | -3,48 | 0,00 | large cell |
| hsa-miR-338-3p | -3,84 | 0,00 | large cell |
| hsa-miR-126 | -3,90 | 0,00 | large cell |
| hsa-miR-451 | -4,24 | 0,00 | large cell |

^a^ microRNAs are listed from highest to lowest fold change differences.

^b^ Upregulation= positive fold change values; downregulation= negative fold change values.
