# Supplementary material for: miR-99a reveals two novel oncogenic proteins E2F2 and EMR2 and represses stemness in lung cancer
Source: Cell Death Dis. 2017 Oct 26;8(10):e3141–. doi: 10.1038/cddis.2017.544 (PMC5680913; doi:10.1038/cddis.2017.544)
Supplement: Supplementary Table 4 [file cddis2017544x6.pdf]

Table S4

miRNA	logqmedian	p (t test)
miR-182	1,455725611	0,008339791
miR-205	1,381675548	0,047293876
miR-210	1,105407944	0,000157653
miR-1246	0,98182623	0,027760992
miR-708	0,975875001	0,002739602
miR-187	0,901783593	0,04451102
miR-155	0,898556642	0,013031686
miR-3141	0,875987956	0,042532507
miR-370	0,854775815	0,007334543
miR-1293	0,835257074	0,027545749
miR-1182	0,814466513	0,0491988
miR-4316	0,811162226	0,02281259
miR-99b*	0,809955725	0,006885568
miR-661	0,770902979	0,00367907
miR-625*	0,718477057	0,011607748
miR-409-3p	0,713219731	0,037282881
miR-200b	0,708976018	0,027125796
miR-615-5p	0,708052624	0,020435298
miR-323-5p	0,705624842	0,010579367
miR-1470	0,685104467	0,034865771
miR-1909*	0,637811481	0,003606877
miR-1250	0,591837572	0,033225317
miR-193b	0,572588869	0,02364827
miR-4295	0,557850248	0,041331921
miR-200a*	0,507229845	0,016139824
miR-668	0,493349414	0,014935894
miR-1273d	0,428350171	0,03732668
miR-939	0,420274849	0,044864572
miR-485-5p	0,420231511	0,018674699
miR-128	0,416807487	0,00365813
miR-183	0,408532792	0,010506902
miR-16-2*	0,388660841	0,012743003
miR-299-3p	0,385622799	0,026533734
miR-4253	0,373119974	0,002253198
miR-1280	0,372441938	0,017184597
miR-512-5p	0,372387012	0,006022655
miR-1266	0,370109431	0,018174183
miR-519a*	0,364105411	0,03504781
miR-200b*	0,331206265	0,004363061
miR-1290	0,323470447	0,032974413
miR-936	0,321736465	0,023489732
miR-1257	0,305942468	0,048134149
miR-9*	0,295299378	0,04462591
miR-518e*	0,282712142	0,037367107
miR-297	0,281596814	0,039522967
miR-1252	0,264103243	0,014647403
miR-874	0,202015522	0,046108778
miR-149*	0,136211058	0,04882373
miR-361-5p	-0,136252175	0,037988804
miR-24	-0,180820666	0,048325867
miR-596	-0,229543548	0,017795191
miR-1277	-0,295106226	0,031734768
miR-518c	-0,320231169	0,02350253
miR-3158	-0,322372138	0,036441618
miR-15a	-0,345207843	0,026056283
miR-136*	-0,347299287	0,042801179
miR-648	-0,385311052	0,046786667
miR-185	-0,415938739	0,029699442
miR-630	-0,420808704	0,039673587
miR-660	-0,425551347	0,03861234
miR-181a	-0,431703268	0,033500473
miR-199b-5p	-0,45169497	0,006834591
miR-1263	-0,458237453	0,044910394
miR-216b	-0,463781638	0,043531086
miR-30c	-0,489507464	0,042107583
miR-103	-0,506538311	0,007948939
miR-139-5p	-0,510691187	0,001106375
miR-3065-5p	-0,526146599	0,017480685
miR-544	-0,53713046	0,031523929
miR-20a*	-0,573006183	0,015707303
miR-338-3p	-0,574737073	0,00866418
miR-26b	-0,594121092	0,020222769
miR-342-3p	-0,601281998	0,009029838
miR-532-5p	-0,604850264	0,002564393
miR-19b-1*	-0,628668345	0,013160969
miR-29c	-0,634650296	0,012299314
miR-15b	-0,663392392	0,021998248
miR-101	-0,734077858	0,004498921
let-7b	-0,746278156	0,049702995
miR-4306	-0,748795556	0,007691412
miR-30b	-0,766338157	0,015307758
miR-3178	-0,773263789	0,014067246
miR-130a	-0,809913863	0,02413579
miR-126	-0,847359807	0,047720145
miR-99a	-0,885004339	0,011872346
miR-140-3p	-0,918230378	0,002188514
miR-497	-0,934276915	0,000620306
miR-30d	-1,064986264	0,025619574
miR-195	-1,160030695	0,000185527
miR-143	-1,257362577	0,000312546
miR-218	-1,346336879	0,000404821
miR-486-5p	-1,48442311	0,002643275
miR-30a	-1,517935222	0,010557763
miR-144	-1,517971535	0,002466894
miR-30a*	-2,089149678	2,47E-05
