# Supplementary material for: miR-99a reveals two novel oncogenic proteins E2F2 and EMR2 and represses stemness in lung cancer
Source: Cell Death Dis. 2017 Oct 26;8(10):e3141–. doi: 10.1038/cddis.2017.544 (PMC5680913; doi:10.1038/cddis.2017.544)
Supplement: Supplementary Table 5 [file cddis2017544x7.pdf]

Histology	Neoadjuvant RT	adyuvante	Relapse	E2F2(total)	EMR2(total)	live-dead	Overall survi	Disease free	E2F2 (positiv	EMR2 (positi	Vimentin	b_Catenin(to	b-catenin (pc	miR-99a
1	1	0	1	0	10	2	10	5	0	0	0	150	0	
1	0	0	1	10	80	1		21	0	0	0	200	1	2,14
3	0	0	1	20	0	1	22	7	0	0	20	200	1	
7	0	1	0	0	0	1			0	0	0		0	
1	0	0	0	0	40	1			0	0	30	35	0	
8	0	0	1	80	20	2	8	7	0	0	80	150	0	
2	0	0	1	0	0	1	34	30	0	0	0	0	0	
1	0	0	1	55	30	2	7	2	0	0	0	150	0	
3	1	0	0	0	0	1			0	0	20	0	0	
6	0	0	0	20	100	1			0	1	0	143	0	6,04
4	0	0	0	270	0	1			1	0			0	
2	0	1	0	10	0	1			0	0	0	0	0	
4	0	0	0	0	0	1			0	0	0	200	1	
1	1	0	0	0	0	1			0	0	0	150	0	
3	0	0	0	120	0	1			1	0		150	0	
2	0	0	0	0	0	2	0		0	0	0	0	0	
3	0	1	1	0	0	1	7	6	0	0	100	0	0	
1	0	0	0	90	115	2	4		1	1	95	80	0	
1	0	0	0	0	20	1			0	0	0	116	0	
2	0	0	0	0	0	1			0	0	0	0	0	
8	0	0	1	0	0	1	22	17	0	0	0	0	0	
8	0	0	1	50	20	2	29	13	0	0	0	0	0	
1	0	0	1	0	0	1	16	11	0	0	20	300	1	
1	0	0	1	270	0	1		15	1	0	10	118	0	
2	0	0	0	0	60	1			0	0	0	0	0	
2	1	0	0	0	0	1	7		0	0	0	0	0	
2	0	0	0	60	50	1			0	0	70	0	0	
2	0	0	0	0	0	1			0	0	0	0	0	
1	0	1	1	50	130	1		10	0	1	0	0	0	
1	0	0	0	90	0	1			1	0	50	300	1	
8	1	1	0	0	10	1			0	0	0	80	0	
1	0	0	1	0	0	1		1	0	0	0	285	1	
1	0	0	0	0	0	1			0	0	0	140	0	
9	0	0	0	100	30	1			1	0	0	110	0	
3	0	0	0	0	0	1			0	0	0	220	1	5,63
2	0	0	0	10	0	1			0	0	0	70	0	
6	0	0	0	0	5	1			0	0	0	200	1	
3	1	0	0	0	30	2	4		0	0	0	225	1	
6	1	0	1	0	0	2	21	6	0	0	0	10	0	7,33
2	0	0	0	0	0	2	2		0	0	0	70	0	4,52
2	0	0	0	0	0	1			0	0	0	100	0	
8	0	1	0	130	120	1			1	1	0	220	1	0,84
3	1	0	1	0	20	1		14	0	0	0	55	0	5,71
1	0	1	1	100	0	1		24	1	0	0	200	1	1,83
2	0	0	0	0	5	1			0	0	10	130	0	4,66
2	0	0	0	0	0	1			0	0	0	140	0	
2	0	0	0	0	0	1			0	0	10		0	
4	0	0	1	100	0	1	19	14	1	0	0	300	1	4,19
1	0	0	0	5	25				0	0	0	125	0	
1	0	0	1	50	160	1		14	0	1	0	200	1	
2	0	0	1	10	0	1	21	12	0	0	0	200	1	1,9
2	1	0	0	0	10	2	3		0	0	20	60	0	
2	0	1	0	0	5	1			0	0		60	0	
2	0	1	0	0	0	1			0	0	70	120	0	
1	0	0	0	100	10	1			1	0	0	180	1	
2	0	0	0	5	0	1			0	0	0	233	1	
3	0	0	1	0	10	1	5	5	0	0	0		0	
2	0	0	1	0	0	1	21	11	0	0	0	125	0	6,81
9	0	1	0	270	0	1			1	0	0	270	1	2,17
2	1	0	1	5	0	1	7	6	0	0	70	240	1	2,89
2	0	0	0	0	5	1			0	0	0	200	1	3,8
4	0	0	0	5	0	1			0	0	0	170	1	5,12
11	0	0	0	100	0	1			1	0	100	230	1	4,49
3	1	0	1	0	0	1		11	0	0		0	0	
9	0	0	0	0	0	1			0	0	0	100	0	2,75
2	0	0	0	0	0	1			0	0	0	120	0	3,37
2	0	0	0	0	0	1			0	0	0	220	1	
1	0	1	0	5	10	1			0	0	60	200	1	
1	0	0	1	0	0	1		16	0	0	50	120	0	
4	0	0	0	10	10	1			0	0	0	80	0	
3	0	0	0	0	0	1			0	0	0	0	0	3,99
1	0	0	0	0	0	1			0	0	0	200	1	
1	0	0	0	110	0	1			1	0			0	
2	0	0	0	0	40	1			0	0	0	25	0	
6	0	0	1	0	10	1		14	0	0	10	180	1	
8	0	0	0	140	180	1			1	1	30	190	1	5,36
2	0	0	0	0	5	2	2		0	0	0	70	0	
8	0	0	0	180	0	2	22		1	0	0		0	
1	0	0	1	0	50	1	16	11	0	0	0	0	0	
2	0	0	0	0	0	1			0	0	0	198	1	4,9
6	0	0	0	0	80	1			0	0	0	151	1	
8	0	0	0	70	0	1			0	0	0	0	0	
2	0	1	0	20	5	1			0	0	0	0	0	
8	0	0	1	0	120	1		3	0	1	20	190	1	6,73
8	0	0	0	0	0	1			0	0	0	60	0	
8	0	0	1	150	0	1	14	8	1	0	0	250	1	4,51
2	1	0	0	10	10	1			0	0	0	0	0	
8	0	1	1	90	100	1	20	15	1	1	0	230	1	2,36
1	0	0	0	0	120	1			0	1	0	300	1	
1	0	0	0	20	130	1			0	1	30	120	0	6,92
1	0	0	1	30	40	1	11	2	0	0	0	200	1	
1	0	0	1	0	0	2	14	11	0	0	0	0	0	
2	0	0	0	20	0	2	2		0	0	80	95	0	
1	1	1	0	230	30	1			1	0	20	200	1	
2	0	0	0	0		1			0		0		0	
6	0	0	0	0	0	1			0	0		100	0	
2	0	0	0	0		1			0				0	
2	0	0	0	0	0	1			0	0	0	0	0	
1	0	0	1	70	0	1		12	0	0			0	
1	0	1	0	30	40	1			0	0	0	80	0	
4	1	0	1	0	0	1		8	0	0	0		0	
3	0	0	0	0	0	2	7		0	0	20	100	0	3,33
8	0	0	1	0	0	1	10	3	0	0	30	100	0	
1	1	0	1	5	135	1	9	7	0	1	20	110	0	5,35
1	0	0	0	0	0	2	2		0	0	0	0	0	
8	0	0	0	0	30	2	5		0	0	0	0	0	
6	0	0	0	0	0	1			0	0	0	300	1	
8	0	0	0	0	10	1			0	0	0	0	0	
1	0	0	1	180	120	1	13	9	1	1	0	200	1	
1	0	0	0	5	160	1			0	1	0	150	0	
8	1	0	1	190	90	1	15	6	1	1	80	230	1	0,39
2	0	0	0	0		1	6		0				0	
2	0	0	1	0	0	1	5	4	0	0	0	0	0	
1	0	0	1	20	60	1	12	4	0	0	0	0	0	
1	0	0	1	10	0	1	19	9	0	0	0	140	0	3,57
1	0	0	0	120	30	1			1	0	40	125	0	
1	0	0	0	0	10	2	1		0	0	0	135	0	
1	0	0	0	150	40	2	15		1	0	60	200	1	
2	0	0	1	0	0	2	8	3	0	0	0			
