# Supplementary material for: miR-99a reveals two novel oncogenic proteins E2F2 and EMR2 and represses stemness in lung cancer
Source: Cell Death Dis. 2017 Oct 26;8(10):e3141–. doi: 10.1038/cddis.2017.544 (PMC5680913; doi:10.1038/cddis.2017.544)
Supplement: Supplementary Table 6 [file cddis2017544x8.doc]

**Supplementary Table 6**. General pathological characteristics of the patients

| Patient number | Disease-free survival ^a^ | Patient status ^b^ | Age | Sex ^c^ | Histological type (T) ^d^ | Differentiation (D) ^e^ |
| --- | --- | --- | --- | --- | --- | --- |
| 1 | 1 | 1 | 61 | M | 1 | 3 |
| 2 | 2 | 0 | 44 | F | 2 | 4 |
| 3 | 2 | 0 | 48 | M | 1 | 2 |
| 4 | 2 | 0 | 68 | M | 1 | 1 |
| 5 | 1 | 1 | 57 | M | 1 | 3 |
| 6 | 1 | 1 | 62 | M | 3 | 2 |
| 7 | 3 | 0 | 75 | F | 1 | 2 |
| 8 | 3 | 0 | 59 | M | 1 | 3 |
| 9 | 3 | 0 | 73 | M | 2 | 4 |
| 10 | 1 | 1 | 66 | M | 2 | 4 |
| 11 | 2 | 0 | 53 | M | 1 | 3 |
| 12 | 2 | 0 | 62 | M | 1 | 3 |
| 13 | 1 | 1 | 74 | M | 3 | 2 |
| 14 | 2 | 0 | 72 | M | 3 | 2 |
| 15 | 1 | 1 | 80 | F | 2 | 4 |
| 16 | 1 | 1 | 75 | M | 1 | 2 |
| 17 | 2 | 0 | 71 | M | 2 | 4 |
| 18 | 2 | 0 | 52 | M | 2 | 4 |
| 19 | 1 | 1 | 70 | M | 3 | 3 |
| 20 | 3 | 0 | 50 | F | 1 | 3 |
| 21 | 2 | 0 | 55 | M | 1 | 3 |
| 22 | 2 | 0 | 55 | M | 2 | 3 |
| 23 | 2 | 0 | 70 | M | 3 | 2 |
| 24 | 1 | 1 | 55 | M | 3 | 2 |

^a^ Disease-free survival (1= death, 2= absence of disease, 3= presence of metastases).

^b^ Patient status (1= death, 2= alive).

^c^ Sex (M= male, F= female).

^d^ Histological type (1= adenocarcinomas; 2= large cell carcinomas; 3= squamous cell carcinomas).

^e^ Degree of differentiation (1 = well differentiated tumors; 2= moderately differentiated tumors; 3 = poorly differentiated tumors; 4= undifferentiated tumors).
