# Supplementary material for: miR-99a reveals two novel oncogenic proteins E2F2 and EMR2 and represses stemness in lung cancer
Source: Cell Death Dis. 2017 Oct 26;8(10):e3141–. doi: 10.1038/cddis.2017.544 (PMC5680913; doi:10.1038/cddis.2017.544)
Supplement: Supplementary Table 7 [file cddis2017544x9.pdf]

Table S7

Primer name	Sequence
F-EMR2	AATGCTAGCCCTTCCTACAAAGACTCCTCCA
R-EMR2	ATCCTGCAGGCCTCGTCATAGTGTACCAGATT
F-E2F2	AGTGCTAGCACAGGGACATTGGACACTAGG
R-E2F2	TTCCTGCAGGTACTAGAACCATGAGGCCTAAGT
Fmir-V-99a	TTGAAACAAAAGCAGTTCGTG
Rmir-V-99a	TTCAGATCCTGGTCCAAACC
