# Supplementary material for: miR-99a reveals two novel oncogenic proteins E2F2 and EMR2 and represses stemness in lung cancer
Source: Cell Death Dis. 2017 Oct 26;8(10):e3141–. doi: 10.1038/cddis.2017.544 (PMC5680913; doi:10.1038/cddis.2017.544)
Supplement: Supplementary Table 7 [file cddis2017544x10.doc]

Fragments for GeneArt Synthesis E2F2 WILD TYPE

Restriction sites: NheI (GCTAGC) / SbfI (CCTGCAGG)

GCTAGCTGAGTGGCCCTGCCTGCCCCCAGCAGCCTGCCCCCGACTCTACCTCCTCACAGACAGGCTGACAGCCCCTCTGCCTGCACAGGGACATTGGACACTAGGTGCTGCCCTCAGGGCATGGGGTCTCCTCGCCTTTCCTGCCCCAGCCGGCAGAAGCTGTGTG**GGGAGATATGAATGGTACGGGTG**AGGAGTGGATAAGGGGTGGTCCTCACCTTCCTAATGGAAGCTGGGCCTAGGGAGGCCCATCCAGTCTTCTGACTTCTGACCTCTCACAAGAAGGCTGCAGGTGAGGTGGCCAAGTCCAGGGAAAGGCCCTGCTACCTCCTTTTGAGGGGTAATTAGGACCCTCGACGTACCAAGAAGCACATAATGCCTTTGTATTTATTTCAGGTTGAGTTGTTTGTTTGTCCTCCCTGAGTTTTAGCAGGGAGGTTGTTCTAGTTTTTAGTGAGACCTCTGCAGACAGGCCCATCACTGTCCATGTTCCAGGGCAGGTCTGGGTTTCCAAGGGAGGGGCCCAGGCTACATCCTTGGTTTCCCCACTGTGGTGGGGGCTGGGACTCTGAGGGGCTGTCCAGTCTGCTAGAATGCTAATTGCACTTAGGCCTCATGGTTCTAGTAAACGGCAGCTGTGGGCCCTTTTGCCTCTTCCCCTGTTCTTGGCCTCACATCTCCAGCTGAGCTGCCGGTCTTGGCTTCCTGGTCGCCTCTGTCCCAGAGATGGTCCCAGGGAGCCATCCTAGGGCAGGTAGCACTGAGGCTCCTGTGGAAACAGGAGCCACCTGCTCAGGAGACCCCTTTCCTGAGGAAGTCCTTACCTCTCCCCTTGAGATGTAAAAATGGTCCAGCAGAGACAAGCTCCCGTGGAAAACAGACAGGAGCATGGGGGCAGCTGTCATGGCTGTGGCGGGCACTTTTCCTCAGAGTTTCTGCCTTGCGCTGGTCCAGGAGCCATTTTGCACCAAGGACTTGGTAGGCAGAGGCAGCCCCACTGTAAAGAAGGGTCAGATTAAAACAAAAAACTGCCAAAAGCATCCCCTCTGCCCCCCATGTGGCACTGGCATCATTCTCTGCTTCCCTGGGAGGAATTTCCTGCAGG

Fragments for GeneArt Synthesis E2F2 **MUTATED**

Restriction sites: NheI (GCTAGC) / SbfI (CCTGCAGG)

GCTAGCTGAGTGGCCCTGCCTGCCCCCAGCAGCCTGCCCCCGACTCTACCTCCTCACAGACAGGCTGACAGCCCCTCTGCCTGCACAGGGACATTGGACACTAGGTGCTGCCCTCAGGGCATGGGGTCTCCTCGCCTTTCCTGCCCCAGCCGGCAGAAGCTGTGTG**GGGAGATATGAATGGTACcaaTG**AGGAGTGGATAAGGGGTGGTCCTCACCTTCCTAATGGAAGCTGGGCCTAGGGAGGCCCATCCAGTCTTCTGACTTCTGACCTCTCACAAGAAGGCTGCAGGTGAGGTGGCCAAGTCCAGGGAAAGGCCCTGCTACCTCCTTTTGAGGGGTAATTAGGACCCTCGACGTACCAAGAAGCACATAATGCCTTTGTATTTATTTCAGGTTGAGTTGTTTGTTTGTCCTCCCTGAGTTTTAGCAGGGAGGTTGTTCTAGTTTTTAGTGAGACCTCTGCAGACAGGCCCATCACTGTCCATGTTCCAGGGCAGGTCTGGGTTTCCAAGGGAGGGGCCCAGGCTACATCCTTGGTTTCCCCACTGTGGTGGGGGCTGGGACTCTGAGGGGCTGTCCAGTCTGCTAGAATGCTAATTGCACTTAGGCCTCATGGTTCTAGTAAACGGCAGCTGTGGGCCCTTTTGCCTCTTCCCCTGTTCTTGGCCTCACATCTCCAGCTGAGCTGCCGGTCTTGGCTTCCTGGTCGCCTCTGTCCCAGAGATGGTCCCAGGGAGCCATCCTAGGGCAGGTAGCACTGAGGCTCCTGTGGAAACAGGAGCCACCTGCTCAGGAGACCCCTTTCCTGAGGAAGTCCTTACCTCTCCCCTTGAGATGTAAAAATGGTCCAGCAGAGACAAGCTCCCGTGGAAAACAGACAGGAGCATGGGGGCAGCTGTCATGGCTGTGGCGGGCACTTTTCCTCAGAGTTTCTGCCTTGCGCTGGTCCAGGAGCCATTTTGCACCAAGGACTTGGTAGGCAGAGGCAGCCCCACTGTAAAGAAGGGTCAGATTAAAACAAAAAACTGCCAAAAGCATCCCCTCTGCCCCCCATGTGGCACTGGCATCATTCTCTGCTTCCCTGGGAGGAATTTCCTGCAGG

Note: A new restriction site for KpnI is designed in the mutant E2F2 sequence

Fragment GeneArt Synthesis EMR2 WILD TYPE

NheI (GCTAGC) / SbfI (CCTGCAGG)

**atgc**GCTAGCTAGAAAAATCTTCTGAATAAGATCTTCCCTCTTTGCCCGTGGAAAATCTGAACAATCTTTGAGCCATCTAGAGGGGAAAGAAAAGACTTTGTTCTGTGTGTTTCAAGAAATTCACCATGTCAGCAATATGAAGGATGTTATGGAAGGCGTGCTAGGCATTCAATTCCTGCAGAAACCGGAAATCTTCCATGCCCTGCAATGTGCTCATCAAACTCTCAGCATATGGACGGCCAGCTGTGGCCCATATCTTGGTCACTCTGAAGCACAATATTTATGAAGCTATAGAACGTTAAGACCTCTTTCACAGCCTCTCCTTCCTACAAAGACTCCTCCAAATCTTAAAATGAAGCAGGAAAACGAGCCTAAGAGGACTTTCATACCGACAACATCTGAAAGGACTAGAATGTTCACACCACGATCTGGATTTCTTAATTTTTTGTTTTTGTTTTT**GTTGTTCTCTAGTTCTACGGGTT**TGATTATTTAGTCATGTGAAAAATATTGATTACTCACACATAGATCAAGAGAGACACGGCTCCTGCCTTCATGGAGCTTTTAGGGGAAAATGAAGTGGCTCTTGCAGCTAGAGTTGACTCAGAAGCCGAAATTCCTAGAAATCAGGTTTCTACTGCTAGGCAATTGAAGTATAAACTATTTTATAAACACTGTCTTCTTTCGTCTTCACACCAACATGCAGAAAAGTTTCTAATCTCAGATCGGGGATGTGCAACAAATTCCATTTCAAAGGAATGACCTGCAAAACTCCTAAATATTCCAAGCAAATGCCCTTAACCCTGTCTGTTATCTGCTTTCCTTGAACAGAAATTCTACATGACCATAAAACCTCGAAGATGGGTATGGCACAGTTCATGCCCTGTAATCCTAGCACTTTGGGAGGGTGAGGCAGGAGGATGGCTCAAGCCCAGGAGTTTGAGACCAGTGTGGGCAACAGAGTGAGAACCATCTCTACCCAAAAAAAAAATTAAAAATTAGCCAAGCATGGTGATGATATAGGAGTTAAGGAGAACCTGCAGG**atgc**

Fragment GeneArt Synthesis EMR2 MUTATED

NheI (GCTAGC) / SbfI (CCTGCAGG)

**atgc**GCTAGCTAGAAAAATCTTCTGAATAAGATCTTCCCTCTTTGCCCGTGGAAAATCTGAACAATCTTTGAGCCATCTAGAGGGGAAAGAAAAGACTTTGTTCTGTGTGTTTCAAGAAATTCACCATGTCAGCAATATGAAGGATGTTATGGAAGGCGTGCTAGGCATTCAATTCCTGCAGAAACCGGAAATCTTCCATGCCCTGCAATGTGCTCATCAAACTCTCAGCATATGGACGGCCAGCTGTGGCCCATATCTTGGTCACTCTGAAGCACAATATTTATGAAGCTATAGAACGTTAAGACCTCTTTCACAGCCTCTCCTTCCTACAAAGACTCCTCCAAATCTTAAAATGAAGCAGGAAAACGAGCCTAAGAGGACTTTCATACCGACAACATCTGAAAGGACTAGAATGTTCACACCACGATCTGGATTTCTTAATTTTTTGTTTTTGTTTTT**GTTGTTCTCTAGTTCTAaGcttTT**TGATTATTTAGTCATGTGAAAAATATTGATTACTCACACATAGATCAAGAGAGACACGGCTCCTGCCTTCATGGAGCTTTTAGGGGAAAATGAAGTGGCTCTTGCAGCTAGAGTTGACTCAGAAGCCGAAATTCCTAGAAATCAGGTTTCTACTGCTAGGCAATTGAAGTATAAACTATTTTATAAACACTGTCTTCTTTCGTCTTCACACCAACATGCAGAAAAGTTTCTAATCTCAGATCGGGGATGTGCAACAAATTCCATTTCAAAGGAATGACCTGCAAAACTCCTAAATATTCCAAGCAAATGCCCTTAACCCTGTCTGTTATCTGCTTTCCTTGAACAGAAATTCTACATGACCATAAAACCTCGAAGATGGGTATGGCACAGTTCATGCCCTGTAATCCTAGCACTTTGGGAGGGTGAGGCAGGAGGATGGCTCAAGCCCAGGAGTTTGAGACCAGTGTGGGCAACAGAGTGAGAACCATCTCTACCCAAAAAAAAAATTAAAAATTAGCCAAGCATGGTGATGATATAGGAGTTAAGGAGAACCTGCAGG**atgc**

Note: A new restriction site for HindIII is designed in the mutant E2F2 sequence
