# Supplementary material for: miR-99a reveals two novel oncogenic proteins E2F2 and EMR2 and represses stemness in lung cancer
Source: Cell Death Dis. 2017 Oct 26;8(10):e3141–. doi: 10.1038/cddis.2017.544 (PMC5680913; doi:10.1038/cddis.2017.544)
Supplement: Supplementary Information [file cddis2017544x11.docx]

**miR-99a reveals two novel oncogenic proteins E2F2 and EMR2 and represses stemness in lung cancer**

Feliciano A^1#^, Garcia-Mayea Y^1#^, Jubierre L^1^, Mir C^1^, Hummel M^2^, Castellvi J^1^, Hernández-Losa J^1^, Paciucci R^1^, Sansano I^1^, Sun Y^1^, Ramón y Cajal S^1^, Kondon H^3^, Soriano A^1^, Segura M^1^, Lyakhovich A^1^ and LLeonart ME^*1^

^1^Biomedical Research in Cancer Stem Cells Group, Pathology Department, Institut de Recerca Hospital Vall d´Hebron (VHIR), Passeig Vall d´Hebron 119-129, 08035 Barcelona, Spain. ^2^Centre for Genomic Regulation, Core Facilities - Microarray Unit, C/ Dr. Aiguader 88, 08003 Barcelona, Spain. ^3^Geriatric unit, Graduate School of Medicine, Kyoto University Hospital, Kyoto, 606-8507, Japan.

# Both authors contribute equally to this work

^*^Corresponding author: Matilde E. LLeonart PhD, Biomedical Research in Cancer Stem Cell Group, VHIR, Passeig Vall d´Hebron 119-129, 08035 Barcelona, Spain; e-mail: matilde.lleonart@vhir.org; Phone: +34-93 4894169; Fax: +34-93 2746808

Running title: miR-99a represses stemness properties

Keywords: microRNAs, miR-99a, E2F2, EMR2, lung cancer, CSCs**Abstract**

Lung cancer is one of the most aggressive tumours with very low life expectancy. Altered microRNA expression is found in human tumours because it is involved in tumour growth, progression and metastasis. In this study, we analyzed microRNA expression in 47 lung cancer biopsies. Among the most downregulated microRNAs we focused on the miR-99a characterization. In vitro experiments showed that miR-99a expression decreases the proliferation of H1650, H1975 and H1299 lung cancer cells causing cell cycle arrest and apoptosis. We identified two novel proteins E2F transcription factor 2 (E2F2) and egf-like module containing, mucin-like, hormone receptor-like 2 (EMR2) downregulated by miR-99a by its direct binding to their 3´-UTR. Moreover, miR-99a expression prevents cancer cells epithelial to mesenchymal transition (EMT) and represses the tumorigenic potential of the cancer stem cells (CSCs) population in these cell lines and mice tumours originated from H1975 cells. The expression of E2F2 and EMR2 at protein level was studied in 119 lung cancer biopsies. E2F2 and EMR2 are preferentially expressed in adenocarcinomas subtypes *versus* other tumour types (squamous and others). Interestingly, the expression of E2F2 correlates with the presence of vimentin and both E2F2 and EMR2 correlate with the presence of β-catenin. Moreover, miR-99a expression correlates inversely with E2F2 and directly with β-catenin expression in lung cancer biopsies. In conclusion, miR-99a reveals two novel targets, E2F2 and EMR2, that are potential oncogenic proteins that play a key role in lung tumorigenesis. By inhibiting E2F2 and EMR2, miR-99a represses in vivo the transition of epithelial cells through an EMT process concomitantly with the inhibition of stemness features and consequently decreasing the CSC population.**Introduction**

Lung cancer is the first leading cause of death worldwide, affecting up to 31% of men and 27% of women ^1^. Differently from other major cancers (e.g., prostate, breast and colorectal carcinomas) that demonstrated significant improvements in 5-year survival during the last 10 years, the 5-year survival rate for lung cancer has remained constant at ~15%. This lack of improvement could be due to the high degree of histological heterogeneity of lung tumours, the difficulties in early diagnosis and the inability to rapidly assess therapeutic effects ^2^. NSCLC accounts for approximately 85% of all lung cancers. In addition, until recently, the subtypes of non-small cell lung cancers (NSCLC): squamous, large cell and adenocarcinoma, were treated similarly, regardless of histological heterogeneity ^3^.

microRNAs have been shown to play an important role in maintaining cellular and physiological homeostasis and to modulate many processes ranging from cellular proliferation, differentiation, metabolism and the regulation of the immune system ^4, 5, 6^. Several microRNAs shown to be deregulated in cancers have been annotated and found to target tumour suppressor genes or oncogenes that play a role in cellular transformation ^7^. Interestingly, different human cancers exhibit different microRNA expression profiles ^8^. However, neither clear correlation between a particular genetic alteration and survival or prognosis has been described, nor there have been any proper genetic markers identified for lung cancer risk so far. Overall, improved knowledge at molecular level is required to assess a reliable treatment response in lung cancer patients.

In the present study, we first screened microRNA expression levels in the normal and tumour tissue of patients with NSCLC using microarrays of microRNAs. We selected a list of microRNAs whose expression pattern were significantly different between normal and cancer tissues. Among the most downregulated microRNAs identified in the biopsies, we focused on miR-99a. miR-99a has been reported to be deregulated in NSCLC and renal cell carcinoma and was shown to play a role in cancer progression ^9, 10^. Interestingly, miR-99a has been associated to the cancer stem (CSC) population in a model of breast cancer but its role in lung CSCs remained unknown ^11^. In this study, we describe two novel target proteins of miR-99a in the lung model: EMR2 and E2F2. Moreover, we show that miR-99a expression is associated with a repression of EMT and stem-cell-genes which are characteristics of aggressive tumours.

**Results**

**A microRNA signature distinguishes normal from tumour tissue in NSCLC patients**

By microRNA signature, we define the list of microRNAs that are differentially expressed in tumors versus normal tissue. Results of the analysis from the microRNA array containing the initial series of 24 patients are shown (supplementary Table 1). We observed significant differences in 97 (38 upregulated and 59 downregulated) out of 799 different microRNAs when comparing normal versus tumour tissues (supplementary Table 2). Based upon the differential expression pattern of the 97 microRNAs found in NSCLC, all 48 samples (24 normal and 24 tumour) were clustered by similarity into subgroups without using any information regarding the identity of the samples. Statistical analyses were performed comparing the expression level of each microRNA in each sample. Samples were significantly grouped into two well-defined normal and cancer groups based on the whole microRNAs contained in platform 1 (supplementary Fig. 1A). However, in a few cases some tumours clustered in the healthy group (possibly due to contamination with normal tissue), and one case of healthy tissue clustered in the tumour group. In order to find a microRNA signature able to define patient subgroups, patients were clustered based on the tumour/normal expression ratios of the 97 selected microRNAs (supplementary Table 2). Significant association between the resulting clusters with tumour type and the degree of tumour differentiation was found (supplementary Fig. 1B and 1C) (*p*< 0.05) *p*= 0.01 and *p*= 0.04 respectively).

No other significant associations were found between the clusters and various clinicopathological features, including age, sex, patient status or disease-free survival, according to the microRNA expression pattern analysis.

In order to identify microRNAs potentially useful as biomarkers to differentiate subtypes of NSCLC, we studied the correlation of the expression of each differentially expressed microRNA (supplementary Table 2) with the histological type. The microRNA miR-205 shows high expression in squamous lung carcinomas (supplementary Fig. 2A) (*p*= 0.02) and microRNAs miR-101, miR-101*, miR-181a, miR-30b and miR-338-3p show significant correlation with the differentiation status of the tumours of NSCLC patients (*p*= 0.04, *p*= 0.04, *p*= 0.03, *p*= 0.03 and *p*= 0.01, respectively) (supplementary Fig. 2B). miR-99a was among of the most downregulated microRNAs (supplementary Table 2). In order to verify the results from the array, a total of 10 patients from Series 1 were studied for the expression of miR-99a by qRT-PCR (supplementary Fig. 3A-B). Results from the qRT-PCR corroborate well the data from the microRNA array for assessing up- or down-regulated miR-99a. Moreover, an independent series of patients (Series 2) was studied for the whole microRNA profile (supplementary Table 3). Ninety-five deregulated microRNAs were identified in this second series of 23 patients (48 upregulated and 47 downregulated), of which 29 microRNAs were common with the first series and miR-99a was also confirmed as one of the most downregulated microRNAs (Fig. 1A and supplementary Table 4). A common signature was established for lung cancer tumours (Fig. 1A). Such 29 deregulated microRNAs in both arrays have the power to distinguish tumor versus normal tissue. The only microRNA able to distinguish cancer subtypes is miR-205 (supplementary Fig. 2A). For further experiments, we focused on the characterisation of miR-99a (Fig. 1B).

**miR-99a reduces the proliferative capacity of lung cancer cell lines**

To assess the potential tumorigenic properties of miR-99a in cancer cells, the H1299, H1650 and H1975 lung cancer cell lines were transduced with miR-99a mimic (miR-99a) or miR-C (non-target control). mRNA levels of miR-99a expression were verified by qRT-PCR (Fig. 2A). Transduction with miR-99a decreased proliferation of all three NSCLC cell lines (Fig. 2B-C and supplementary Fig. 4A). Proliferation curves of NSCLC cancer cells stably expressing miR-99a were similar to the transient expression experiments previously observed for the H1299, H1650 and H1975 cells (supplementary Fig. 4B-D, and data not shown). In order to verify the suppressive function of miR-99a at physiological levels, the above cell lines were treated with 1:10 and 1:3 diluted viral supernatant of transfected phoenix cells and compared with undiluted counterparts (Fig. 2). Note that even at maximal dilution (1:10), miR-99a was able to to suppress cell proliferation (supplementary Fig. 5). miR-99a levels at different dilutions is shown (supplementary Fig. 6A). Conversely, anti-miR-99a was applied to determine whether it was able to reverse proliferative effect of miR-99a. Indeed, the expression of anti-miR-99a promoted cell proliferation (Fig. 2D and data not shown).

**miR-99a suppresses tumorigenicity by inducing apoptosis and cell cycle arrest**

To uncover possible mechanisms of the miR-99a-mediated suppression of cell proliferation, cell cycle arrest, apoptosis and senescence were studied with cells transiently expressing miR-99a. An increase in apoptosis was detected after miR-99a expression in the three cell lines (Fig. 2E). Moreover, the presence of apoptosis was accompanied with cell cycle arrest in miR-99a expressing cells compared to control cells (Fig. 2F). H1299 and H1650 accumulated in G2 and H1975 in G1 phase of the cell cycle. Accordingly, a decrease in the number of miR-99a expressing cells was confirmed by Tripan-blue staining (supplementary Fig. 4E). miR-99a expressing cells were negative for β-galactosidase staining, discarding the possibility of cells entering senescence (data not shown). Moreover, cells infected with miR-99a viral construct at 1:10 dilution were also able to increase apoptosis in lung cancer cells (supplementary Fig. 6B and data not shown). Overall, a tumour-suppressor function of miR-99a was observed upon both transient and stable expression of miR-99a in all lung cancer cell lines.

**E2F2 and EMR2 are revealed as two novel miR-99a targets**

By performing bioinformatic search for possible targets of miR-99a, EMR2 and E2F2 were revealed as two potential novel targets. A schematic representation of the binding site of miR-99a with EMR2 and E2F2 is shown (Fig. 3A). Western-blot analysis confirmed the downregulation of both EMR2 and E2F2 proteins upon expression of miR-99a (Fig. 3B and supplementary Fig. 7A). To validate these proteins as miR-99a targets, the 3´-UTR of each gene was cloned in the pmir-GLO vector (Fig. 3A). Both the pmir-GLO3´-UTR-E2F2 and pmir-GLO3´-UTR-EMR2 plasmids were transfected in HEK293T cells concomitantly with miR-99a or miR-C as previously described ^12^. The presence of miR-99a was able to inhibit by ~2 fold the expression of each 3´-UTR indicating that these two proteins are targets of miR-99a (Fig. 3C). Moreover, the effect of an anti-miR-99a was accompanied by an increase of expression of EMR2 and E2F2 proteins (Fig. 3D and supplementary Fig. 7B). Overall these data provide compelling evidence of two novel targets for miR-99a. Lastly, in order to test the contribution of E2F2 and EMR2 to cell proliferation, siRNA depletion of E2F2 and EMR2 was performed (supplementary Fig. 7D). The depletion of E2F2 and/or EMR2 in H1299, H1650 and H1975 cells significantly reduced cell proliferation (Fig. 3E).

**miR-99a suppresses invasion and migration while favours adhesion of lung cancer cell lines**

Two previous publications described the ability of miR-99a to reduce migration and invasion in T24 and EJ bladder cancer cell lines and A540 and H1299 lung cancer cell lines ^13, 14^. In order to address if miR-99a reduces migration and invasion, H1299, H1975 and H1650 cells were transduced with miR-99a and both transwell mobility and wound healing assays were performed. As revealed, miR-99a significantly reduced the migration and invasion in the three cancer cell lines (Fig. 2G, supplementary Fig. 8A, supplementary Fig. 9A-B, and data not shown). Moreover, miR-99a overexpression stimulated cell adhesion (supplementary Fig. 8B and data not shown). In order to validate the miR-99a biological effect, the anti-miR-99a was included for comparison (Fig. 2G, Fig. 8A and supplementary Fig. 9A). In addition, for the above three cell lines a partial contribution of E2F2 and EMR2 to migration, invasion and adhesion was confirmed by siRNA experiments (Fig. 3F, supplementary Fig. 8C-D and supplementary Fig. 9C). These results support the tumour-suppressor function of miR-99a and its link with the identified targets.

**E2F2 and EMR2 overexpression is concomitant to miR-99a**

In order to analyze whether E2F2 or EMR2 expression was able to rescue the suppressive function of miR-99a, co-expression of E2F2 gene or EMR2 gene was performed concomitantly with miR-99a in the indicated cell lines (Fig. 4A). We demonstrate E2F2 but not EMR2 was able to rescue the suppressive function of miR-99a in H1299, H1650 and H1975 cells (Fig. 4A). We also tested if concomitant expression of E2F2 and EMR2 with miR-99a modulates migration. Both E2F2 and EMR2 were able to rescue significantly the inhibitory role of miR-99a on cell migration (Fig. 4B). Importantly, E2F2 showed a potent effect on cell migration as the sole expression of E2F2 significantly increased migration in comparison with control cells (miR-C).

**miR-99a suppresses tumorigenicity in vivo**

To confirm the tumour-suppressor function of miR-99a in vivo, 1 x 10^6^ miR-99a expressing H1975 cells and controls were xeno-injected subcutaneously to immunocompromised mice. The levels of miR-99a and target proteins were verified after transfection and just before the injection (Fig. 5A and data not shown). Tumours formed by miR-99a were significantly smaller (p< 0.05) than those formed in control group (Fig. 5B-D). The microscopic examination of tumours revealed a consistent pattern of heterogeneous tumours with fusocellular morphology in the control group in contrast to a more homogeneous epithelial pattern observed in tumours over-expressing miR-99a (Fig. 5E, see arrows). The morphology of the tumours in the control group resembled the EMT transition associated with more aggressive tumour phenotypes ^15^. Increased protein expression levels of N-Cadherin and decreased E-cadherin expression, as indicators of the EMT, were observed in the mice tumours derived from the control group but not in those originated frommiR-99a expression (Fig. 5F). Moreover, changes in protein expression of N-Cadherin and E-cadherin were also observed in H1975 cells expressing miR-99a versus control cells (supplementary Fig. 10A).

Other genes related to the EMT, such us Snail and Twist, were also found significantly downregulated in both the miR-99a expressing mice tumours and NSCLC cell lines but not in human biopsies (Fig. 6A-C). In addition, downregulation of the stem cell genes Nanog, Oct3/4 and Sox2 were consistently observed in the mice tumours, NSCLC cell lines and human biopsies (Fig. 6D-F).

**miR-99a expression targets CSCs**

Since the CSC-related genes were significantly downregulated in the mice tumours, NSCLC cells and human biopsies, we hypothesised that miR-99a might play an important role in acquisition of CSC features in lung tumorigenesis. In order to detect cells with a stem cells properties, the “Side Population” (SP) discrimination assay has been performed for H1975 cells expressing miR-99a or miR-C. The percentage of the SP detected in control or miR-99a expressing H1975 cells was 2.66% and 1.02%, respectively, suggesting increased stem-like cancer cells (Fig. 7A). Expression of miR-99a also decreased two times the SP numberin H1299 cells (Fig. 7B). We then studied contribution of E2F2 and EMR2 proteins to the formation of SP. The siRNA deletion of E2F2 and EMR2 resulted in the decrease of the SPin H1975 and H1299 cells by 0.62% and 0.52% versus 1.30% and 0.87% and 0.93% versus 2.34% respectively (supplementary Fig. 11A and B). In order to validate the biological activity of the remaining population of SP, three different functional assays were performed. First, H1299, H1650 and H1975 cells -that normally grow in standard adherent conditions- were transfected with miR-99a and forced to grow in tri-dimensional cultures (soft-agar) allowing to form colonies during 10-15 days. The miR-99a expressing cells formed fewer colonies than control cells (Fig. 7C). Second, in order to validate the self-renewal abilit, H1299, H1650 and H1975 cells were transfected with miR-99a and transferred to non-adherent plates with a stem cell media. NSCLC spheres were dissociated and re-seeded again under the same conditions and the process repeated up to three consecutive generations (G3). Evidence that the spheroid cellshold some properties of cancer stem cells under these conditions was proven by increase expression of stem cell genes ALDH1, Nestin, Sox4 and Oct4 in comparison to the same cells growing in adherent conditions (supplementary Fig. 10B). Under these conditions, miR-99a expressing CSCs formed fewer colonies than those CSCs derived from control cells (Fig. 7D). In order to observe if the miR-99a target proteins E2F2 and EMR2 were involved in the ability of CSCs to form spheres, we tested performed siRNA experiments. The depletion of either protein decreased the ability of corresponding NSCLC to form spheres with siRNA-E2F2 showing a major effect (Fig. 7E). Concomitant expression of E2F2 or EMR2 with miR-99a restored the effect of sphere formation (Fig. 4C). Third, in order to test the known resistance of CSCs to conventional chemotherapy, miR-99a-expressing spheroid (CSCs), adherent (parental) or control cells were grown in the presence of CDDP and cell viability was measured after 48h post-treatment. CSCs revealed higher resistance to CDDP than their corresponding parental counterparts (Fig. 7F). However, we observed that miR-99a expression sensitised CSCs to the exposure of CDDP but has no effect on the parental H1975 cells (Fig. 7F). To observe if EMR2 and E2F2 were involved in such CSCs sensitisation, we siRNA depleted each protein and then treated the cells with CDDP. Partial contribution of each siRNA to such sensitisation was significantly observed in parental cells but not in CSCs (Fig. 7G). Lastly, representative lung cancer biopsies were assessed for the SP percentage and miR-99a expression. An inverse correlation with miR-99a level was found (Fig. 7H and supplementary Fig. 11C). Overall, the above results suggest that miR-99a reverses the CSC phenotype by decreasing their tumorigenic potential.

**E2F2 and EMR2 proteins are expressed in a subset of lung cancer patients**

In order to test if E2F2 and EMR2 are involved in lung cancer progression, immunohistochemistry (IHC) analyses from 119 patients were performed (Fig. 8A and supplementary Table 5). Expression of E2F2 was detected in 22 of 119 patients (18%) and EMR2 in 14 of 119 patients (11.7%). Concomitant expression of E2F2 and EMR2 occurs in a subset of lung cancer samples (Fig. 8B). E2F2 and EMR2 expression occurs predominantly in the patients with adenocarcinoma, rather than with squamous cell carcinoma (Fig. 8C-D). To determine if E2F2 and/or EMR2 expression was able to inversely correlate with miR-99a expression in lung cancer biopsies, RNA was extracted from a group of 30 randomly taken patients out of 119. The expression level of E2F2 (but not EMR2) inversely correlated with miR-99a expression (Fig. 8). To determine if the expression of E2F2 and EMR2 proteins correlated with the presence of the EMT, vimentin expression was analysed (Fig. 8A and supplementary Table 5). E2F2 expression significantly correlated with vimentin expression (Fig. 8F). Moreover, the expression levels of β-catenin were studied as its potential link with Wnt pathway activation ^16, 17^ -known to be active in CSCs (Fig. 8A and supplementary Table 5). Expression of β-catenin inversely correlated with miR-99a expression (Fig. 8G). In addition, β-catenin expression correlated with the expression of E2F2 and EMR2 (Fig. 8H and 8I). These results suggest that over-expression of E2F2 and potentially EMR2 can be associated to lung cancers which would pursue through an EMT with potential activation of stemness.

**Discussion**

In this study, a microRNA signature that revealed microRNA candidates of oncogenic and tumour suppressor functions in lung cancer is proposed. The results from the array indicate that, in general, cancer can be distinguished from healthy tissue based on the microRNA expression profile. Particularly, miR-99a, could be a potential therapeutic marker in lung cancer, as previously proposed ^18^. In this article, we described two novel miR-99a targets: E2F2 and EMR2 (also known as ADGRE2 or CD312), representing two oncogenic proteins that can modulate tumour suppression in NSCLCs. Downregulation of these proteins by miR-99a provokes apoptosis and cell cycle arrest with a consequent decrease of cancer cells proliferative capacity. We found that miR-99a-mediated decrease of cell proliferation elicits a different response depending on the cell line. Thus, G2/M cell cycle arrest is clearly induced in H1299 and H1650 cells, and G0/G1 cell cycle arrest is induced in H1975 cells upon miR-99a expression. This finding highlights the functional plasticity of miR-99a according to the cellular context. Our results support previous studies reporting a tumour suppressive function for miR-99a as a general mechanism for other cancer models besides^19^.

E2Fs members represent a family of transcription factors involved in a myriad of functions, including the control of cell cycle. However, the specific function of E2F2 is not very clear. For example, some studies in mice have shown anti-proliferative function of E2F2 ^20, 21^, while other reports have described a pro-proliferative function ^22, 23, 24, 25^.

EMR2, a member of the EGF-TM7 receptor family, is a cell-surface receptor involved in cell attachment to proteoglycans in a Ca2^+^-dependent manner ^26^. EMR2 has an important role in myeloid cell migration and trafficking by binding to cell-surface molecules (i.e., sulphated chondroitin chains). The altered composition of proteoglycans in the stroma of invasive tumours may be involved in the attraction or function of tumour-associated leukocytes resulting in important pro- or anti- tumour effects ^27^. Interestingly, a significant but low number of colorectal carcinomas are positive for EMR2 and EMR2 is relevant in breast cancer progression ^28, 29^. In NSCLC cells, we found that the action of both E2F2 and EMR2 concurred to the suppressor function of miR-99a, thereby supporting a proliferative and pro-oncogenic role for these proteins in cancer. In fact, the inhibition of E2F2 and EMR2 reduced proliferation, migration and invasion and increased adhesion. Moreover, depletion of E2F2 and EMR2 reduced the number of colonies in three-dimensional cultures. Accordingly, E2F2, but not EMR2 overexpression concomitantly to miR-99a was able to rescue the suppressive function of miR-99a in proliferation of lung cancer cells. Instead, both E2F2 and EMR2 were able to partially rescue NSCLC cell migration and the ability to form spheres in our model. We conclude from these experiments that the major contributor of the suppressive function of miR-99a in lung cancer cell lines is E2F2 and the contribution of EMR2 is only partial. These results are in agreement with the described role of EMR2 in migration and adhesion ^28, 30^. The minor effect of EMR2 to rescue the suppressive function of miR-99a could be related to the fact that EMR2 has up to 13 different isoforms and it is not known the contribution of each one to the final function of the protein ( in our experiments we are using the isoform 1).

On the other hand, it has been proposed that EMT is a process by which epithelial cells loose their contact with neighbouring cells to become mesenchymal stem cells. EMT promotes contact inhibition and is associated with the initiation of metastases and chemoresistance ^31, 32^. There are two contradictory studies that associate the role of E2F2 and EMT. For example, it has been described that upregulation of E2F2 is associated with a delay in contact inhibition ^33^. On the contrary, Fujiwara et al. showed that E2F2 is associated to EMT in the specific case of Myc-induced tumours in a model of breast cancer ^21^. In the same line of evidence the same authors further showed that loss of E2F2 sharply increased the percentage of lung metastasis in MMTV-Myc mice ^34^. Interestingly, we found that all mice tumours formed by miR-99a overexpression showed a fusocellular pattern different from the tumours formed in the control group, supporting a role of miR-99a in EMT inhibition concomitant to a downregulation of stem-cell-genes. We also recognized that miR-99a levels in the mice tumors was able to persist upon few weeksfrom the initial transient transfection in H1975 cells(supplementary Fig. 11D). Of notice, lung cancer biopsies with high expression levels of miR-99a showed downregulation of the stem-cell-genes when compared to biopsies with very low expression. The consistency of the downregulation of the stem-cell-genes in the cell lines, mice tumours and human biopsies, lead us to hypothesise that miR-99a could modulate the CSC population. Lung cancer cells overexpressing miR-99a have less number of CSCs and less self-renewal ability, which are known characteristics of CSCs. Our results support a recent study showing that E-cadherin repression increased the amount of CSCs in A549 lung cancer cells ^35^. Our findings support that an activation of the EMT process is linked to an increase in CSCs number, as shown in a mice model and human tumours. Moreover, our data suggest that miR-99a inhibits the viability of the CSC population in lung cancer cells, being E2F2 and EMR2 particularly relevant in this process. Moreover, miR-99a exerts a tumour-suppressor function not only in CSCs (decreasing their percentage and functionality) but also in parental cells from the lung (decreasing their proliferation capacity). In particular, CSCs sensitisation to CDDP by miR-99a is not only due to the inhibition of E2F2 and/or EMR2 and more proteins might be involved.

Moreover, we found a positive correlation between the expression of E2F2 and EMR2 in human tumours. In a representative number of lung cancer biopsies from patients, miR-99a expression inversely correlated with E2F2 expression. Particularly in adenocarcinomas E2F2 expression also correlated with vimentin, a marker associated with EMT phenotype.

Activation of Wnt/β-catenin pathway has been associated to increased lung tumour metastasis and proliferation ^36, 37^. Differential β-catenin expression levels and localization are associated with cancer prognosis ^38^. We observed that: i) miR-99a expression inversely correlated with β-catenin expression and ii) E2F2 and EMR2 expression levels correlated with β-catenin expression in the lung cancer biopsies. This association supports the results obtained from the mice tumours that pointed out that lower expression of E2F2 and EMR2 proteins (due to miR-99a upregulation), favours an epithelial phenotype and downregulation of stemness-associated genes.

To our knowledge, this is the first study that associates the tumour suppressor function of miR-99a with E2F2 and EMR2 repression. Its action is accompanied by a decrease of EMT and downregulation of stem-cell-genes *in vivo*. In view of our data, we propose that those lung cancer tumours with high miR-99a levels and corresponding repression of E2F2 and EMR2 would evolve more favourably due to an inhibition of cell proliferation. This process is accompanied by avoiding epithelial cancer cells through an EMT process that would render fewer number and functionality of CSCs.

**Patients and methods**

**Patients**

Forty-seven paired samples of human NSCLC and their matched adjacent non-cancerous tissues were collected at the time of surgery between 2008 and 2010 from the Tumour Bank of the Pathology Department of the Hospital Vall d´Hebron. The matched normal tissues were obtained 5 cm from the tumour margin, which were further confirmed by pathologists. Upon resection, human surgical specimens were immediately frozen in liquid nitrogen and stored at -80°C in the refrigerator. Patients recruited for this study did not undergo any therapy before resection. Series 1 contained 24 patients and Series 2, 23 patients. Series 1 includes: 11 adenocarcinomas, 7 large cell and 6 squamous cell lung carcinomas. The characteristics of the patients from Series 1 are shown (supplementary Table 6). Series 1 was used for screening of the microRNA expression profiling using the facility from the Centre for Genomic Regulation (CRG platform, version 4.0 AFM). For each patient, the following pathological and clinical parameters were studied: Disease-free survival, patient status (dead, alive), sex, age (<60 versus >60), histological type (squamous, large cell, adenocarcinomas) and degree of differentiation (well, moderate, poor, undifferentiated). In order to corroborate the results from the array, a second series (Series 2) was analysed with a different microRNA platform and different company (FEBIT, version 15.0 Geniom Biochip MPEA). A third series of 119 patients (series 3) was chosen for protein study by IHC. All procedures used in the present study have been approved by the Ethics Committee of Hospital Vall d’Hebron.

**RNA Extraction**

Normal and tumour frozen tissue from the lung was used for RNA extraction from a total of 47 patients. H&E staining of the slides from frozen biopsies was performed to ensure that the tissue area would have an adequate tumour density (<60%). Total RNA was isolated with a MirVana kit (Ambion, Austin, TX) according to the manufacturer’s instructions. RNA quality was assessed by Bioanalyser (RIN ratio > 8). RNA from lung cancer cell lines was extracted with the same protocol from subconfluent culture dishes (ø= 10cm).

**Microarray preparation**

Human microRNA microarrays (G4470B; Agilent, Santa Clara, CA) containing 13,737 probes corresponding to 799 microRNAs and 22 control probes, were hybridised with RNA from normal and cancer tissue of 24 NSCLC patients. Briefly, 500 ng of total RNA from each sample were chemically labeled by dephosphorylation using Calf Intestinal Alkaline Phosphatase (CIP) and ligated to Cyanine3-pCp with a T4-RNA ligase using Agilent miRNA Complete Labeling and Hyb Kit (p/n5190-0456, Agilent). Labeled samples were dried, resuspended in 18 μl of nuclease-free water and treated with in situ hybridisation buffer for 20 h at 55°C. Samples were then washed at room temperature for 5 min in Gene Expression Wash Buffer 1 (Agilent) and 5 min at 37ºC in Gene Expression Wash Buffer 2 (Agilent). The images were generated on a confocal microarray scanner G2565BA (Agilent) at 5 μm resolution and quantified using Feature Extraction (Agilent).

### **Real-time PCR**

Quantitative real-time PCR was used to determine levels of miR-99a (Hs04231437_s1), miR-205 (ID 000509), Nanog (Hs04399610_g1), Oct3/4 (Hs04260367_gH), Sox2 (Hs01053049_s1), Snail (Hs00161904_m1), Twist (Hs01675818_s1) and housekeeping genes: U6 (ID 0001093), TBP (Hs00427620_m1) and IPO8 (Hs00183533_m1) using the Assays-on-Demand Taqman Gene Expression Assays (Applied Biosystems, Foster City, CA) according to the procedure previously described ^39^. qRT-PCR was performed to determine the relative mRNA levels of: 1) miR-99a in 10 cancer tissues (versus normal) to corroborate the array data, 2) miR-99a mRNA levels in H1299, H1975 and H1650 NSCLC cell lines (control and transfected with miR-99a), 3) Nanog, Oct3/4, Sox2, Snail and Twist genes in the H1299, H1975 and H1650 NSCLC cell lines expressing miR-99a versus control, 4) Nanog, Oct3/4, Sox2, Snail and Twist genes in H1975 mice tumours formed by miR-99a expression (versus control), 5) Nanog, Oct3/4, Sox2, Snail and Twist genes in 8 cancer tissues: the top 4 with highest miR-99a expression (Patients 6, 26, 28 and 48 from Series 1) versus the top 4 with the lowest miR-99a expression (Patients 14, 18, 36 and 40) from the array (Series 1). Results were conducted in triplicate in at least three independent experiments.

**Bioinformatics search**

Potential miR-99a targets were predicted and analysed by using publicly available algorithm-based databases, including PicTar (<http://pictar.mdc> berlin.de/), TargetScan (http://www.targetscan.org/), miRanda (http://www.microrna.org/), and DIANA-microT (<http://diana.cslab.ece.ntua.gr/>). To select the putative miR-99a mRNA targets, we focused on those detected in more than one miRNA database. Of these, E2F2 and EMR2 were selected.

**Plasmid construction**

For the miR-99a stable overexpression experiments, premiR-99a was cloned into retroviral vector miR-V, which was kindly donated by Dr. R. Agami (Netherlands Cancer Institute, Amsterdam). Approximately 500 nt of the genomic DNA sequence that encodes for primary miR-99a and its natural flanking sequences was selected for PCR amplification, according to a previously described procedure ^12^. Primers are detailed in supplementary Table 7.

For performing luciferase assay, cloning to pmir-GLO pDNA (Promega) containing the luciferase reporter and renilla gene has been performed with 1088 bp 3´-UTR-E2F2 mRNA sequence (NCBI Reference Sequence: NM_004091.3) artificially synthesized (ThermoFisher) and 1024 bp 3´UTR-EMR2 (ADGRE2) (NCBI Reference Sequence: NM_013447.3) artificially synthesized (ThermoFisher). For the mutant E2F2 miR-99 construct, the seeding sequence was replaced with GGGAGATATGAATGGTACcaaTG having recognition site for KpnI restriction enzyme (Fig. 3A and supplementary Table 8). For the mutant EMR2 miR-99 construct the seeding sequence was replaced with GTTGTTCTCTAGTTCTAaGcttTT having recognition site for HindIII restriction enzyme (Fig. 3A and supplementary Table 8). All the subcloned inserts had NheI (3´GCTAGC5´) and SbfI (3´CCTGCAGG5´) restriction sites. In all cases, the cloned PCR products were validated by sequencing (data not shown).

**Protein analysis**

Total cell lysates were prepared from a subconfluent 10-cm dish. Cells were washed in PBS and lysed in 1 ml of lysis buffer (50 mM Tris-HCl, pH 7.5, 1% NP-40, 10% glycerol, 150 mM NaCl, plus 2 mM complete protease inhibitor cocktail) (Roche Diagnostics, Barcelona, Spain). From each sample, 50 μg of protein, quantified with the Bio-Rad protein assay (Bio-Rad, Hercules, CA, USA), was analysed by gel electrophoresis on a 6–12% SDS polyacrylamide gel and transferred onto a nitrocellulose membrane. The following antibodies were used for western blot analysis: E2F2 (sc632, Santa Cruz), EMR2 (sc34334, Santa Cruz), E-cadherin (#3195, Cell Signaling), N-Cadherin (ab18203, Abcam) and β-actin (A2228, Sigma-Aldrich). In all cases, membranes were incubated overnight at 4°C with the primary antibodies in T-TBS with 5% nonfat dry milk. The membranes were then washed with T-TBS and incubated with horseradish peroxidase-conjugated anti-mouse or anti-rabbit secondary antibody (Sigma-Aldrich). After additional washes with T-TBS, the antigen-antibody complexes were visualised with an enhanced chemiluminescence kit (Millipore).

**Cell culture**

H1975, H1299 and H1650 NSCLC cell lines were obtained from the American Type Culture Collection (ATCC). The three cell lines were grown in RPMI-1640 medium (Lonza). Media was supplemented with 10% FBS (Sigma), 100 U/ml penicillin, and 100 μg/ml streptomycin. All of the cells were grown at 37°C in a humidified incubator with 5% CO_2_. Cells were passaged regularly twice per week and maintained at subconfluence.

**Transfection-Transduction**

Transient transfection of H1299, H1650 and H975 cells using Lipofectamine 2000 (Thermo Fisher Scientific) or jetPEI (Polyplus) was performed with the synthetic precursors of miR-99a called pre-miR-99a or miR-99a mimic (designated here as miR-99a) (ID: AM17100; Ambion), anti-miR-99a (ID: 10719; Ambion), a Cy3 dye labeled negative control (ID: AM17020; Ambion) or negative control miR-C (ID: AM17110; Ambion). H1299, H1650 and H1975 cells were seeded at 2.5 × 10^5^ and 2.0 × 10^5^ and 1.5 cells × 10^5^ per well, respectively, in 6-well plates and transiently transfected with miR-99a or anti-miR-99a to a final concentration of 80 nM with lipofectamine (Thermo Fisher Scientific) according to manufacturer’s instructions. Seventy two hours after transfection, cells were counted and the cellular lysates were collected for analysis of the protein expression of the selected putative miR-99a targets. Transfection efficiency was determined by fluorescence microscopy and compared to Cy3 dye-labeled negative control.

To study the transient effects of EMR2 or E2F2 silencing, H1299, H1650 and H1975 cells were seeded at 2 × 10^5^ cells per well in a 6-well plate and transfected the following day with the indicated siRNAs or controls (NT-siRNA or scramble -Sc-) using lipofectamine. After 48h, cells were counted and lysed for RNA and protein extraction as previously described ^40^.

For the stable expression, miR-99a (cloned in miR-V –designated miRV-99a), miR-V and miRV-GFP were included, the latter for checking infection efficiency that in all cases was ~100%. Thirty μg of each retroviral vector were transfected into Phoenix cells in 10-cm culture plates with FuGENE (Roche). The viral particles in the supernatant were harvested 48 h after transfection and used to infect H1299, H1650 and H1975 cells. Each stably transduced cell line was selected with blasticidin (10 μg/ml) for 12 days. In all cases, parental cells (uninfected) were maintained and treated with blasticidin for checking appropriate selection. Results were conducted in triplicate in at least three independent experiments.

For the preparation of the analysis of the SP cells, H1299 and H1975 cells were transduced using Lipofectamine 2000 concomitantly with Cy3 dye-labeled negative control plus miR-99a versus miR-C (1:10 ratio) in order to select only transfected cells.

**Growth curves**

H1975, H1299, and H1650 cells were seeded at 1 × 10^6^ cells per 10-cm plate. Parental cells for each cell line, the control cells (miRV-GFP-infected), and cells that expressed miR-99a (miRV-99a) were grown simultaneously. Every 3 days, the cells from each cell line were counted and reseeded at a density of 1 × 10^6^ cells per 10-cm plate, as indicated by the 3T3 protocol described ^12^. In addition, 5 × 10^4^ cells per well were reseeded every 3 days in 24-well plates in triplicate and fixed. Cell staining was performed with crystal violet. For Cisplatin (CDDP) (Sigma-Aldrich) treatment, H1975 cells were tritated at different concentrations and 20 µM were chosen. Cells (1 x 10^3^) were seeded in 96 well-plates by quintuplicate and treated the next day with CDDP. Cells were fixed and measured for viability with the MTS assay after 48 h of treatment. Results were conducted in triplicate in at least three independent experiments.

**Luciferase reporter assay**

The luciferase experiments were performed in HEK293T cells. HEK293T cells were seeded at 1 × 10^4^ cells per well in a 96-well plate and were transfected the following day with Lipofectamine 2000 (Invitrogen) with the following molecules: the synthetic miRNA precursor miR-99a (ID: AM17100; Ambion), and negative control miR-C (ID: AM17110; Ambion), Cy3 (ID: AM17020) and the pmir-GLO plasmid (Promega) containing the luciferase reporter and also the renilla gene (control) versus pmirGLO3’-UTR-E2F2 or pmirGLO3’-UTR-EMR2. The transfection efficiency was approximately 95%, and luciferase activity was measured 48 h after transfection with the dual luciferase reporter assay as described by the manufacturer (Promega). In each case, the miR-99a concentrations were measured by titrating the miR-99a with each pmirGLO3’-UTR mRNA construct to establish a dose-response relationship between 10–80 nM (data not shown). Results were conducted in triplicate in at least three independent experiments.

**Cell cycle analysis**

For the cell cycle analysis, a fluorescence-activated cell sorting Calibur flow cytometer (FACS Calibur, Becton Dickinson, E0772; BD Biosciences, San Jose, CA, USA) was used to analyse H1299, H1650 and H1975 cells that transiently or stably expressed miR-99a versus negative control. One million cells from each sample were fixed in 70% ethanol for 15 min at -20°C, treated with 100 μg/ml RNase A (Sigma-Aldrich), and stained with 5 μg/ml of propidium iodide (PI) (Sigma-Aldrich). For each sample, 2 × 10^4^ cells were analysed, and the percentage of cells in each phase of the cell cycle was calculated based on the DNA content determined with FACS Express Software. Results were conducted in triplicate in at least three independent experiments.

**Annexin V-APC apoptosis analysis**

H1299, H1650 and H1975 cells were transfected with miR-99a versus control and infected with miRV-99a or miRV-GFP and were selected with blasticidin for stable expression. The quantification of apoptotic cells was performed with the Annexin V-APC Detection Kit (eBioscience) according to the manufacturer’s instructions. The samples were then analysed by FACS. For each sample, 2 × 10^4^ cells were analysed and the results were analysed with the FACS Express Software. The results were confirmed in at least three independent experiments.

**Animal model**

Mouse from NMRI-FOXn1 nu/nu strain (Janvier) were used for xeno-injection. H1975 lung cancer cells (1 x 10^6^ cells) were transiently transfected with 100 nM of control microRNA (miR-C, non-silencing control) or miR-99a mimic. Thirty-six hours later, viable cells were harvested and injected subcutaneously (1 x 10^6^/mouse) in the flank of 6 week-old female NMRI-FOXn1 nu/nu in 300 μl of PBS and matrigel (1:1). H1975 cells were inyected into a total of 16 mice (8 mice were xeno-injected with control microRNA and 8 mice with miR-99a). Tumour volume was measured every 2–3 days during two weeks using an electronic calliper. At the respective scheduled surgery, mice were euthanised and tumours removed and weighted. Tumours were then fixed in 10% formalin, paraffin-embedded, and 5 μm sections were H&E-stained. A portion of each tumour was snap-frozen and reserved for RNA and protein studies.

**Analysis of SP cells**

SP analysis was performed as described with slight modifications ^41^. Briefly, NSCLC cells were digested with TrypLE Express (Gibco), washed with PBS and resuspended at a density of 1 x 10^6^ cells/ml in pre-warmed RPMI-1640 culture medium (Gibco) supplemented with 2% FBS and 10 mM 4-(2-hydroxyethyl)-1-piperazineethanesulfonic acid (HEPES) (Sigma Aldrich). Then, the cells were incubated at 37˚C for 120 minutes with 5 µg/ml Hoechst 33342 dye (Thermo Scientific). Control cells were incubated with 50 µM verapamil (Sigma‑Aldrich) for 15 min at 37˚C prior to the addition of Hoechst dye to validate the SP detection (data not shown). For cell dead discrimination, PI (Sigma Aldrich) at 5 µg/mL was added to the cells prior to FACS analysis. Cell samples were analysed and sorted using FACSAria II flow cytometer (BD Biosciences).

**IHC**

The expression of E2F2 (sc-632, Santa cruz), EMR2 (sc-34334, Santa cruz), β-catenin (#9562, Cell signaling) and vimentin (V6389, Sigma-Aldrich) proteins were studied by IHC in the 119 patient samples (series 3) with lung cancer. β-catenin was located at the cell membrane, E2F2 and vimentin at cytoplasmic level, and EMR2 nuclear as described ^28^. Paraffin-embedded biopsies were included in tissue microarrays (TMAs), as previously described ^12^. The sections were incubated at RT during 2 h with the indicated antibodies and the immunostaining were performed using the ChemMate DAKO EnVision Detection Peroxidase/DAB kit (Dako). Quantification of the reaction was performed using the histoscore system, as previously described ^12^.

**Statistical Analysis**

All sample data were analysed using SPSS software (version 11.0, SPSS), and *p*< 0.05 was considered statistically significant (**p*< 0.05; ***p*< 0.01). To identify differentially regulated microRNAs, moderated paired t-tests were applied using limma ^42^. Pairwise differences between groups were analysed using the Student t test (proliferation curves, spheroids formation, mRNA study by qRT-PCR, protein staining by IHC, cell cycle profiles, apoptosis, migration, invasion and adhesion). All data described in this manuscript, will be shared with the scientific community upon request.

**Conflict of interest**

The authors declare no conflict of interest.

**Acknowledgements**

We are very grateful to R. Somoza and T. Moliné for his technical assistance and to the Tumour Bank of the Pathology Department (Hospital Vall d´Hebron, Barcelona) for providing the tumour samples analysed herein. We are very grateful to Jauma Comás from the Parc Cientific at the IRBB for the FACS assistance. We are very grateful to Dr. Hsi-Hsien Lin (Chang Gun University, Taiwan) for the EMR2 cDNA plasmid and Dr. Ana Maria Zubiaga (Universidad del Pais Vasco, Bilbao, España) for giving us the E2F2 cDNA plasmid.

**Funding**

This work was supported by grants from the Instituto de Salud Carlos III, grants PI12/01104 and PI15/01262 cofinanced by the European Regional Development Fund (ERDF) (ME LLeonart); ME. LLeonart is a FIS (Fondo de Investigación Sanitario, Health Ministry) investigator (CP03/00101). Feliciano A is granted by the FIS. And Garcia-Mayea is granted with a VHIR fellowship. Lyakhovich A is granted by Marie Curie INCOMED senior fellowship, contract grant number GA267128 .

**References**

1. Torre LA, Siegel RL, Ward EM, Jemal A. Global Cancer Incidence and Mortality Rates and Trends--An Update. *Cancer Epidemiol Biomarkers Prev* 2016, **25:** 16-27.

2. Torre LA, Siegel RL, Jemal A. Lung Cancer Statistics. *Adv Exp Med Biol* 2016, **893:** 1-19.

3. Borczuk AC, Toonkel RL, Powell CA. Genomics of lung cancer. *Proc Am Thorac Soc* 2009, **6:** 152-158.

4. Mendell JT. miRiad roles for the miR-17-92 cluster in development and disease. *Cell* 2008, **133:** 217-222.

5. Dalmay T. MicroRNAs and cancer. *J Intern Med* 2008, **263:** 366-375.

6. Poy MN, Eliasson L, Krutzfeldt J, Kuwajima S, Ma X, Macdonald PE*, et al.* A pancreatic islet-specific microRNA regulates insulin secretion. *Nature* 2004, **432:** 226-230.

7. Zhang B, Pan X, Cobb GP, Anderson TA. microRNAs as oncogenes and tumor suppressors. *Dev Biol* 2007, **302:** 1-12.

8. Lu J, Getz G, Miska EA, Alvarez-Saavedra E, Lamb J, Peck D*, et al.* MicroRNA expression profiles classify human cancers. *Nature* 2005, **435:** 834-838.

9. Cui L, Zhou H, Zhao H, Zhou Y, Xu R, Xu X*, et al.* MicroRNA-99a induces G1-phase cell cycle arrest and suppresses tumorigenicity in renal cell carcinoma. *BMC Cancer* 2012, **12:** 546.

10. Gao W, Shen H, Liu L, Xu J, Xu J, Shu Y. MiR-21 overexpression in human primary squamous cell lung carcinoma is associated with poor patient prognosis. *J Cancer Res Clin Oncol* 2011, **137:** 557-566.

11. Yang Z, Han Y, Cheng K, Zhang G, Wang X. miR-99a directly targets the mTOR signalling pathway in breast cancer side population cells. *Cell Prolif* 2014, **47:** 587-595.

12. Feliciano A, Castellvi J, Artero-Castro A, Leal JA, Romagosa C, Hernandez-Losa J*, et al.* miR-125b acts as a tumor suppressor in breast tumorigenesis via its novel direct targets ENPEP, CK2-alpha, CCNJ, and MEGF9. *PLoS One* 2013, **8:** e76247.

13. Wu D, Zhou Y, Pan H, Zhou J, Fan Y, Qu P. microRNA-99a inhibiting cell proliferation, migration and invasion by targeting fibroblast growth factor receptor 3 in bladder cancer. *Oncol Lett* 2014, **7:** 1219-1224.

14. Chen C, Zhao Z, Liu Y, Mu D. microRNA-99a is downregulated and promotes proliferation, migration and invasion in non-small cell lung cancer A549 and H1299 cells. *Oncol Lett* 2015, **9:** 1128-1134.

15. Ye X, Weinberg RA. Epithelial-Mesenchymal Plasticity: A Central Regulator of Cancer Progression. *Trends Cell Biol* 2015, **25:** 675-686.

16. Zhang J, Jiang HY, Zhang LK, Xu WL, Qiao YT, Zhu XG*, et al.* C-FLIPL modulated Wnt/beta-catenin activation via association with TIP49 protein. *J Biol Chem* 2016.

17. Williams KE, Bundred NJ, Landberg G, Clarke RB, Farnie G. Focal adhesion kinase and Wnt signaling regulate human ductal carcinoma in situ stem cell activity and response to radiotherapy. *Stem Cells* 2015, **33:** 327-341.

18. Sun M, Hong S, Li W, Wang P, You J, Zhang X*, et al.* MiR-99a regulates ROS-mediated invasion and migration of lung adenocarcinoma cells by targeting NOX4. *Oncol Rep* 2016, **35:** 2755-2766.

19. Hu Y, Zhu Q, Tang L. MiR-99a antitumor activity in human breast cancer cells through targeting of mTOR expression. *PLoS One* 2014, **9:** e92099.

20. Opavsky R, Tsai SY, Guimond M, Arora A, Opavska J, Becknell B*, et al.* Specific tumor suppressor function for E2F2 in Myc-induced T cell lymphomagenesis. *Proc Natl Acad Sci U S A* 2007, **104:** 15400-15405.

21. Fujiwara K, Yuwanita I, Hollern DP, Andrechek ER. Prediction and genetic demonstration of a role for activator E2Fs in Myc-induced tumors. *Cancer Res* 2011, **71:** 1924-1932.

22. Baiz D, Dapas B, Farra R, Scaggiante B, Pozzato G, Zanconati F*, et al.* Bortezomib effect on E2F and cyclin family members in human hepatocellular carcinoma cell lines. *World J Gastroenterol* 2014, **20:** 795-803.

23. Wang H, Zhang X, Liu Y, Ni Z, Lin Y, Duan Z*, et al.* Downregulated miR-31 level associates with poor prognosis of gastric cancer and its restoration suppresses tumor cell malignant phenotypes by inhibiting E2F2. *Oncotarget* 2016, **7:** 36577-36589.

24. Bai H, Harmanci AS, Erson-Omay EZ, Li J, Coskun S, Simon M*, et al.* Integrated genomic characterization of IDH1-mutant glioma malignant progression. *Nat Genet* 2016, **48:** 59-66.

25. Nguyen-Vu T, Vedin LL, Liu K, Jonsson P, Lin JZ, Candelaria NR*, et al.* Liver x receptor ligands disrupt breast cancer cell proliferation through an E2F-mediated mechanism. *Breast Cancer Res* 2013, **15:** R51.

26. Lin HH, Chang GW, Davies JQ, Stacey M, Harris J, Gordon S. Autocatalytic cleavage of the EMR2 receptor occurs at a conserved G protein-coupled receptor proteolytic site motif. *J Biol Chem* 2004, **279:** 31823-31832.

27. Lewis SM, Treacher DF, Edgeworth J, Mahalingam G, Brown CS, Mare TA*, et al.* Expression of CD11c and EMR2 on neutrophils: potential diagnostic biomarkers for sepsis and systemic inflammation. *Clin Exp Immunol* 2015, **182:** 184-194.

28. Davies JQ, Lin HH, Stacey M, Yona S, Chang GW, Gordon S*, et al.* Leukocyte adhesion-GPCR EMR2 is aberrantly expressed in human breast carcinomas and is associated with patient survival. *Oncol Rep* 2011, **25:** 619-627.

29. Aust G, Hamann J, Schilling N, Wobus M. Detection of alternatively spliced EMR2 mRNAs in colorectal tumor cell lines but rare expression of the molecule in colorectal adenocarcinomas. *Virchows Arch* 2003, **443:** 32-37.

30. Huang YS, Chiang NY, Hu CH, Hsiao CC, Cheng KF, Tsai WP*, et al.* Activation of myeloid cell-specific adhesion class G protein-coupled receptor EMR2 via ligation-induced translocation and interaction of receptor subunits in lipid raft microdomains. *Mol Cell Biol* 2012, **32:** 1408-1420.

31. Wrighton KH. Cell migration: EMT promotes contact inhibition of locomotion. *Nat Rev Mol Cell Biol* 2015, **16:** 518.

32. Fischer KR, Durrans A, Lee S, Sheng J, Li F, Wong ST*, et al.* Epithelial-to-mesenchymal transition is not required for lung metastasis but contributes to chemoresistance. *Nature* 2015, **527:** 472-476.

33. Liu X, Tseng SC, Zhang MC, Chen SY, Tighe S, Lu WJ*, et al.* LIF-JAK1-STAT3 signaling delays contact inhibition of human corneal endothelial cells. *Cell Cycle* 2015, **14:** 1197-1206.

34. Yuwanita I, Barnes D, Monterey MD, O'Reilly S, Andrechek ER. Increased metastasis with loss of E2F2 in Myc-driven tumors. *Oncotarget* 2015, **6:** 38210-38224.

35. Farmakovskaya M, Khromova N, Rybko V, Dugina V, Kopnin B, Kopnin P. E-Cadherin repression increases amount of cancer stem cells in human A549 lung adenocarcinoma and stimulates tumor growth. *Cell Cycle* 2016, **15:** 1084-1092.

36. Nguyen DX, Chiang AC, Zhang XH, Kim JY, Kris MG, Ladanyi M*, et al.* WNT/TCF signaling through LEF1 and HOXB9 mediates lung adenocarcinoma metastasis. *Cell* 2009, **138:** 51-62.

37. Yamada HY, Kumar G, Zhang Y, Rubin E, Lightfoot S, Dai W*, et al.* Systemic chromosome instability in Shugoshin-1 mice resulted in compromised glutathione pathway, activation of Wnt signaling and defects in immune system in the lung. *Oncogenesis* 2016, **5:** e256.

38. Gao ZH, Lu C, Wang MX, Han Y, Guo LJ. Differential beta-catenin expression levels are associated with morphological features and prognosis of colorectal cancer. *Oncol Lett* 2014, **8:** 2069-2076.

39. Artero-Castro A, Callejas FB, Castellvi J, Kondoh H, Carnero A, Fernandez-Marcos PJ*, et al.* Cold-inducible RNA-binding protein bypasses replicative senescence in primary cells through extracellular signal-regulated kinase 1 and 2 activation. *Mol Cell Biol* 2009, **29:** 1855-1868.

40. Artero-Castro A, Perez-Alea M, Feliciano A, Leal JA, Genestar M, Castellvi J*, et al.* Disruption of the ribosomal P complex leads to stress-induced autophagy. *Autophagy* 2015, **11:** 1499-1519.

41. Goodell MA, Brose K, Paradis G, Conner AS, Mulligan RC. Isolation and functional properties of murine hematopoietic stem cells that are replicating in vivo. *J Exp Med* 1996, **183:** 1797-1806.

42. Smyth GK. Linear models and empirical Bayes methods for assessing differential expression in microarray experiments. *Statistical applications in genetics and molecular biology* 2004, **3:** article 3.

**Figure legends**

**Figure 1** microRNA array study. (*A*) Schematic representation of the methodology to identify a microRNA signature able to distinguish healthy from tumour tissue in NSCLC patients is shown. The signature comprises 29 microRNAs: 9 upregulated (red) and 20 downregulated (green) in comparison with normal tissue). (*B*) Array results for the miR-99a which was chosen out of the whole de-regulated microRNAs for future experiments. N, normal tissue; T, tumour tissue; N_p_, pool of normal tissues; T_p_, pool of tumour tissues; FC, fold change, *p* (T-test value).

**Figure 2** miR-99a exerts a tumour suppressor function in lung cancer cells. (*A*) Relative qRT-PCR shows the expression levels of NSCLC cell lines upon miR-99a transient expression. (*B*) Proliferation curves of miR-99a expressing cells versus control cells (transfection time was considered 0 h). (*C*) Cristal violet stained colonies indicative of cell proliferation of miR-99a expressing cells and controls after 48 h upon transfection. (*D*) Cell number of the indicated cells transfected with an anti-miR-99a or control (miR-C) microRNA. (*E*) Plots of miR-99a expressing cells and controls stained with Annexin-V indicative of apoptotic cells and analysed by FACS. Note that miR-99a increases late apoptosis (right upper panel) in all cases. (*F*) Cell cycle profile of miR-99a cells versus controls in the indicated cell lines. Note that miR-99a arrests cells in G2 phase of the cell cycle in H1299 and H1650 cell lines and arrests cells in G1 in H1975 cells. PI, propidium iodide. Data are representative of at least three independent experiments. (*G*) Relative migration of H1299, H1650 and H1975 cells transduced with miR-99a and anti-miR-99a. (*p< 0.05; **p< 0.01).

**Figure 3** E2F2 and EMR2 are downregulated upon miR-99a expression. (*A*) Schematic representation indicating the cloned region of the 3´-UTR of EMR2 (1046bp) and E2F2 (1102) mRNA. Positions of the binding regions of miR-99a (corresponding to the seed sequence) to each 3´-UTR are indicated (463-469 and 173-179 respectively). The asterisks in the 3´-UTR region indicate the changes introduced in the DNA sequence for the design of each respective mutant. (*B*) Western-blots showing the protein levels of E2F2 and EMR2 in miR-99a expressing cells in the indicated cell lines. β-actin is shown for protein content. (*C*) Luciferase reporter experiment for the miR-99a expressing cells concomitantly with the pmir-GLO plasmid (control), the pmirGLO3´-UTR-E2F2 or the pmirGLO3´-UTR-EMR2. Relative expression of Renilla versus Firefly (luciferase) expression is shown. Note that miR-99a is able to decrease the expression of both 3´-UTRs. (*D*) Western-blots of E2F2 and EMR2 in anti-miR-99a expressing cells in the indicated cell lines. Data are representative of at least three independent experiments. (*E*) Cell number of H1299, H1650 and H1975 cells transduced with the indicated siRNAs and counted upon 48 h from transfection. (*F*) Relative migration of H1299, H1650 and H1975 cells transduced with the indicated siRNAs. (*p< 0.05; **p< 0.01).

**Figure 4** E2F2 and EMR2 genes overexpression concomitantly to miR-99a. (*A*) Proliferation curves of the indicated cancer cell lines upon 48h from transfection with the indicated genes and/or miR-99a. Right panel corresponds to EMR2 and left panel corresponds to E2F2 expressing cells (with and without miR-99a). Note the total proliferative rescue upon E2F2 expression and lack of rescue of EMR2 under the same conditions. (*B*) Relative migration of H1299 and H1975 cells transduced with miR-99a and E2F2 or EMR2. Significant increase in migration occurs upon overexpression of E2F2 and EMR2 concomitantly to miR-99a when compared to miR-99a alone (plus empty vector). (*C*) Number of spheroids from third generation (G3) cells growing in stem-cell-media and non-adherent dishes upon overexpression of E2F2 and EMR2 concomitantly to miR-99a *versus* controls.

**Figure 5** miR-99a expression delayed tumour formation in vivo. (*A*) H1975 cells were transiently transduced with miR-99a and measured for miR-99a RNA levels by qRT-PCR before injecting in mice. (*B*) Graph showing tumour volume in miR-99a expressing tumours versus controls. (*C*) Representative pictures of tumours from mice transduced with miR-99a versus controls. (*D*) Graph showing tumour weight of miR-99a expressing tumours versus controls. (*E*) Example of phenotypic morphology of mice tumours (H & E staining) expressing miR-99a or control microRNA. Arrows indicate the fusocelular pattern (control group) or epithelial pattern (miR-99a group) in each case. (*F*) Western-blots showing expression of N-Cadherin and E-Cadherin for the indicated group of mice tumours.

**Figure 6** Relative mRNA levels of genes related to stemness. Snail and Twist genes were analysed by qRT-PCR in the mice tumours (*A*), NSCLC cell lines (*B*) and human tumours (*C*). Nanog, Oct3/4 and Sox2 genes were analysed by qRT-PCR in the mice tumours (*D*), NSCLC cell lines (*E*) and human tumours (*F*). (*C*, *F*) From the array results (supplementary Table 1), top 4 cancers with higher expression of miR-99a were selected (designated as “High”) and the 4 cancers with the lowest expression of miR-99a (designated as “Low”) are depicted in the graph.

**Figure 7** miR-99a modulates stemness. (*A*, *B*) Plots from the FACS analysis of the SP cells representative of CSCs in miR-99a expressing cells versus controls in the indicated cell lines (H1975 and H1299 respectively). Note the decrease in SP in miR-99a expressing cells after 48 h upon transfection. (*C*) Number of spheroids from the indicated cells expressing miR-99a or control microRNA growing in soft-agar (as representative of three-dimensional culture). (*D*) Number of spheroids from third generation (G3) CSCs growing in stem-cell-media and non-adherent dishes in miR-99a expressing cells *versus* controls. (*E*) Number of spheroids from third generation (G3) CSCs (growing as in Fig. 6D) in the indicated cells depleted for E2F2 and EMR2 versus controls. (*F*) Survival of CSCs (growing as in Fig. 6D) and parental cells to CDDP treatment in miR-99a expressing cells and controls. (*G*) Survival of CSCs (growing as in Fig. 6D) and parental cells to CDDP treatment in cells depleted for E2F2 and EMR2. Data are representative of at least three independent experiments. (*H*) Example of two lung cancer biopsies for the analysis of the percentage of SP cells. See mRNA levels of miR-99a for comparison (Fig. S8C).

**Figure 8** E2F2, EMR2, vimentin and β-catenin expression as revealed by IHC. (*A*) Representative pictures of the staining of the indicated proteins in lung cancer patients. (*B*) Significant correlation between E2F2 and EMR2 (*p*= 0.026). (-) negative expression for the indicated proteins; (+) positive expression for the indicated proteins. (*C*, *D*) Correlation of E2F2 and EMR2 with adenocarcinoma tumours (1, adenocarcinoma; 2, squamous cell carcinoma; 3, others uncommon tumour types from the lung such as neuroendocrine, etc.) (*p*= 0.003 and *p*= 0.01 respectively). (*E*) Significant correlation of miR-99a and E2F2 protein expression (*p*= 0.025). (*F*) Significant correlation of E2F2 and vimentin protein expression (*p*= 0.003). (*G*) Significant correlation of miR-99a and β-catenin protein expression (*p*= 0.035). (*H*, *I*) Significant correlation of E2F2 and EMR2 with β-catenin protein expression in the 119 patients analysed (*p*= 0.001 and *p*= 0.032, respectively).
