# Supplementary material for: miR-99a reveals two novel oncogenic proteins E2F2 and EMR2 and represses stemness in lung cancer
Source: Cell Death Dis. 2017 Oct 26;8(10):e3141–. doi: 10.1038/cddis.2017.544 (PMC5680913; doi:10.1038/cddis.2017.544)
Supplement: Supplementary Table Legends [file cddis2017544x12.doc]

**Supplementary Table 1**

Whole array of microRNAs (CRG platform, version 4.0 AFM) showing the expression at RNA level of the indicated microRNAs. The results correspond to Series 1. FC, Fold change; p, p value (with/without adjustment).

**Supplementary Table 3**

Whole array of microRNAs (FEBIT platform, version 15.0 Geniom Biochip MPEA) showing the expression at RNA level of the indicated microRNAs. The results correspond to Series 2.

**Supplementary Table 5**

Pathological characteristics of the patients, expression levels of the indicated proteins by IHC and RNA levels of miR-99a. The results correspond to Series 3.

**Supplementary Table 7**

Sequence of the indicated primers used for this study

**Supplementary Table 8**

Synthetic DNA fragments corresponding to the 3´-UTR of the indicated genes used for the cloning into the pmiR-Glo plasmid.
